# The crucial prognostic signaling pathways of pancreatic ductal adenocarcinoma were identified by single-cell and bulk RNA sequencing data

**DOI:** 10.1007/s00439-024-02663-4

**Published:** 2024-03-25

**Authors:** Wenwen Wang, Guo Chen, Wenli Zhang, Xihua Zhang, Manli Huang, Chen Li, Ling Wang, Zifan Lu, Jielai Xia

**Affiliations:** 1 Department of Health Statistics, School of Military Preventive Medicine, Ministry of Education Key Lab of Hazard Assessment and Control in Special Operational Environment, Fourth Military Medical University, Xi’an, 710032 Shaanxi Province China; 2 Shaanxi Provincial Key Laboratory of Infection and Immune Diseases, Shaanxi Provincial People’s Hospital, Xi’an, 710068 Shaanxi Province China

## Abstract

Pancreatic ductal adenocarcinoma (PDAC) is a malignant tumor with poor prognosis and high mortality. Although a large number of studies have explored its potential prognostic markers using traditional RNA sequencing (RNA-Seq) data, they have not achieved good prediction effect. In order to explore the possible prognostic signaling pathways leading to the difference in prognosis, we identified differentially expressed genes from one scRNA-seq cohort and four GEO cohorts, respectively. Then Cox and Lasso regression analysis showed that 12 genes were independent prognostic factors for PDAC. AUC and calibration curve analysis showed that the prognostic model had good discrimination and calibration. Compared with the low-risk group, the high-risk group had a higher proportion of gene mutations than the low-risk group. Immune infiltration analysis revealed differences in macrophages and monocytes between the two groups. Prognosis related genes were mainly distributed in fibroblasts, macrophages and type 2 ducts. The results of cell communication analysis showed that there was a strong communication between cancer-associated fibroblasts (CAF) and type 2 ductal cells, and collagen formation was the main interaction pathway.

**Supplementary Information:**

The online version contains supplementary material available at 10.1007/s00439-024-02663-4.

## Introduction

Pancreatic ductal adenocarcinoma (PDAC) is a highly malignant, aggressive and dismal solid tumor. Although immunotherapy has improved the treatment of tumors and the prognosis of patients in recent years, the prognosis of PDAC has not changed significantly, with a 5-year survival rate of only 8% (Siegel et al. 2023). The poor prognosis is mainly related to high heterogeneity, difficult early diagnosis, and limited efficacy account for the unfavorable prognosis (Park et al. 2021). Accurate early diagnosis and radical resection can significantly improve the prognosis of patients, but the current serum markers, mainly including car-bohydrate antigen 19–9 (CA19-9) (Lee et al. 2020) and carcinoembryonic antigen (CEA) (Hammarström 1999), have limited specificity and accuracy for early screening of patients with PDAC. Improving the accuracy of prognostic assessment can provide clinical decision support for doctors and patients. Therefore, there is an urgent need to identify potential prognostic and predictive biomarkers as well as intervenable signaling pathways that could precisely stratify patients by developing an effective prognostic prediction model.

The development of bioinformatics and multi-omics databases provides convenience for exploring the expression patterns of malignant tumors and constructing prognostic and diagnostic models (Ma et al. 2022). Using next-generation sequencing (NGS), gene mutations and alterations in molecular pathways can be identified and used to develop strategies to selectively kill cancer cells such as *KRAS*, *NRG1*, *NTRK* and related molecules in PDAC patients (Jones et al. 2019; Waters and Der 2018; Xie et al. 2022). Despite the promising predictive power observed in the aforementioned studies, these prognostic signatures are based on bulk RNA-seq, which cannot detect the exact cellular and molecular changes in tumor cells, as it mainly focuses on the "average" expression of all cells in a sample. At the same time, more and more evidence show that the occurrence and development of tumors largely depends on the complex microenvironment in which they are located, including tumor cells and their surrounding immune cells, cancer-associated fibroblasts and endothelial cells (Xiao and Yu 2021). However, bulk RNA-seq only shows the average expression level of the whole tissue, which may have resulted in a bias for individual tumor cells. scRNA-seq can reveal differentially expressed genes between tumor cells and normal ductal cells without interference from pancreatic tissue stroma and immune cells. It should be noted that single-cell sequencing results are limited to low sequencing depths, and some differential genes may not be detected. In addition, single-cell sequencing is costly, and there are currently far fewer single-cell datasets with prognostic information available for PDAC than batch sequencing (Moncada et al. 2020). Therefore, integrating the results of bulk-seq and single-cell data to improve the resolution of the source of tumor heterogeneity on the basis of maximizing the discovery of differential genes is a better approach for PDAC heterogeneity analysis and prognostic modeling.

In this study, we aimed to develop and validate a prognostic PDAC model based on scRNA-seq and bulk-seq datasets, and further analyze the expression patterns of the model genes in single-cell data to further explore the intratumor heterogeneity and possible pathways of PDAC from the survival results. Unicox regression and lasso regression were used to further screen variables, and finally 12 genes were selected for the multivariate cox regression model. The model was externally validated in three independent data sets and found to have good discrimination and calibration. Cytological tests revealed that the expression levels of most prognostic genes exhibited an increase with the progression of cell malignancy, except for *SERPINB5*, *IL22RA1*, *MPZL2*, and *S100A14*. In the analysis of differential gene expression, gene mutation profile and immune infiltration between high- and low-risk groups, it was found that the expression of macrophages and monocytes was significantly different, and the low-risk group was more sensitive to chemotherapy drugs. Finally, the analysis of prognostic gene expression and intercellular communication in single cells revealed that the prognostic difference between the high- and low-risk groups may be mediated by the collagen formation pathway.

## Materials and methods

### Data acquisition and preprocessing

10 × scRNA-seq data of 24 PDAC samples were downloaded from the PRJCA001063 series (https://ngdc.cncb.ac.cn/bioproject/browse/PRJCA001063), which included in a total of 41,986 cells. Transcriptome sequencing data, mutation data, and corresponding clinical information of pancreatic adenocarcinoma (PAAD) were obtained from the TCGA database (https://tcga-data.nci.nih.gov/tcga/). Datasets (GSE62165, GSE71989, GSE16515, GSE91035, GSE62452, GSE57495) were downloaded from Gene Expression Omnibus (GEO) (https://www.ncbi.nlm.nih.gov/geo) and ICGC-CA dataset was downloaded from the ICGC database (https://dcc.icgc.org). PDAC data from the above dataset were screened for downstream analysis. The sample size and basic situation of data acquisition are shown in Supplementary Table 1. The transcript sequencing data of TCGA, read counts data of ICGC, and series matrixes data of GEO datasets were processed by “log2 (data + 1).”, which were conducted by R.

### scRNA-seq analysis

All specimens were merged as an original seurat object using Seurat (version 4.2.0) R toolkit (Satija et al. 2015). This object was filtered to remove unqualified cells (< 200 genes/cell, > 20% mitochondrial genes, transcripts/cell < 1000 or > 20 000) and genes (< 10 cells/gene) and was normalized (LogNormalize). The percentage of mitochondria genes and total counts were used to scale data. Next, 2000 highly variable genes were selected for PCA. The ‘harmony’ method was used to integrate the dataset from different specimens. Significant principal components were identified by JackStraw analysis. Cell atlas was visualized using t-SNE analysis.

The cell type of each cluster was identified by aligning marker genes to known signature genes reported in previous studies and CellMarker2.0 database (http://biocc.hrbmu.edu.cn/CellMarker2.0/) (Hu et al. 2023). The known signature genes were *AMBP*, *CFTR*, *MMP7* (ductal cell 1); *KRT19*, *KRT7*, *TSPAN8*, *SLPI* (ductal cell 2); *PRSS1*, *CTRB1*, *CTRB2*, *REG1B* (acinar); *CHGB*, *CHGA*, *INS*, *IAPP* (endocrine cell); *RGS5*, *ACTA2*, *PDGFRB*, *ADIRF* (stellate cell); *LUM*, *DCN*, *COL1A1*(fibroblast), *CDH5*, *PLVAP*, *VWF*, *CLDN5* (endothelial cell); *AIF1*, *CD64*, *CD14*, *CD68* (macrophage), *CD3D*, *CD3E*, *CD4*, *CD8* (T cell), *MS4A1*, *CD79A*, *CD79B*, *CD52* (B cell).

### Differentially expressed genes (DEGs) screening based on scRNA-seq and GEO datasets

TCGA queue data and single cell data were integrated and analyzed by “Scissor” algorithm to obtain the cell subsets that were most and least correlated with prognosis. The “FindMarkers” algorithm in the “Seurat” package was used to identify differential genes between the most and least relevant cell subsets. We selected the gene expression data of GSE62165, GSE71989, GSE16515, and GSE91035 and divided the data into the tumor group and the control group, respectively. “limma” R package (version 3.50.3) (Phipson et al. 2016) was used to perform the differential expression analysis of genes between two groups at first. Genes with a corrected *P*-value < 0.05 and |log fold change (FC)|> 1 were considered DEGs. The “RobustRankAggreg” (RRA) R package (version 1.2.1) was used to integrate all DEGs ranked by logFC, and 234 up-regulated DEGs and 101 down-regulated DEGs were finally obtained for subsequent analysis.

### Model construction and validation

Univariate Cox (Unicox) regression analysis was performed to examine the association between DEGs and patients’ survival time. DEGs with a *P* value less than 0.05 were identified using the Wald ratio test. In order to further narrow down the candidate genes, we applied the least absolute shrinkage and selection operator (LASSO) algorithm to prevent model overfitting (Zhang et al. 2022). Multivariate Cox regression analysis was applied to screen for genes independently related to survival at the same time. The prognostic models constructed by the candidate genes obtained from the two screening strategies were compared, and the 12 signature genes that were finally used for modeling were identified. All genes were checked to meet the assumption of equal proportional hazards using the “cox.zph” function. All TCGA patients were randomly divided into training (*n* = 119) and internal validation (n = 51) cohorts according to the proportion of 7:3. The prognostic formula used was as follows:

1 $$Risk score= \sum_{i=1}^{n}{\beta }_{i}\times {exp}_{i}$$

where ${\beta }_{i}$ represents coefficients in the multivariate Cox analysis and ${exp}_{i}$ is gene expression value. According to the optimal threshold of ROC curve, all patients were divided into high-risk group and low-risk group. Survival curves and risk plots were generated by the R software “survminer” (version 0.4.9) and the “ggrisk” (version 1.3) package to visualize survival differences and status for each patient. The receiver operating characteristic (ROC) curve was drawn by the R software “timeROC” (version 0.4) package to evaluate the predictive effect of the risk score on the 1-, 3-, and 5-year OS of PDAC patients. Then, TCGA validation set and PACA_CA, GSE62452, GSE57495 were employed for the internal and external validations of the prognostic model. In addition, we established nomogram based on risk factors and independent prognostic factors to predict the risk and OS, and 1- and 3-year survival prediction calibration curves were drawn using the R package “rms” (version 6.3.0) to characterize the discrimination of the prognostic model.

### Analyses of signature genes

We first analyzed the expression of signature genes between high and low risk groups in different datasets, and then analyzed the correlation of marker gene expression by spearman method. Protein–protein interaction (PPI) analysis of signature genes was performed base on the STRING online database (https://string-db.org) which integrates the interaction information of multitudinous proteins. Cytoscape (version 3.7.1) was used to customize and analyze the PPI network. The cytoHubba app (Chin et al. 2014) in Cytoscape was used for calculating the hub signature genes by MCC algorithm.

### Functional enrichment analysis

Using the criteria |log2FC|≥ 0.8 and FDR < 0.05, we screened for DEGs of the two risk groups. The functions of DEGs were annotated by Gene Ontology (GO) and Kyoto Encyclopedia of Genes and Genomes (KEGG) using R packages “clusterProfiler” (version 4.2.2) and “org.Hs.eg.db” (version 3.14.0).

### Mutation status analysis

The sample mutation data in the TCGA cohort was extracted, and then the mutation status between two risk subgroups was analyzed by the R package “maftools” (version 2.10.05).

### Assessment of tumor immune cell infiltration

Single-sample gene set enrichment analysis (ssGSEA) was used to explore the differences of immune cell subtypes. The gene list of key factors involved in tumor immune regulation was obtained from Tracking Tumor Immunophenotype (hrbmu.edu.cn). The expression levels of negatively regulated genes between high and low-risk groups in four datasets, including TCGA, GSE62452, GSE57495, and ICGC, were analyzed (Xu et al. 2018). Meanwhile, we further analyzed the differential expression of immune checkpoint-related genes between high and low risk groups.

Accumulating evidence suggested that tumor immune microenvironment played an important role in development of cancers. In order to set up the association of the estimated proportion of immune and stromal with signature genes expression, we used R package “estimate” (version 1.0.13) to estimate the ratio of immune-stromal component in TME. In addition, results were exhibited in the form of these three kinds of scores: ImmuneScore, StromalScore, and ESTIMATEScore. The higher score estimated in ImmuneScore or StromalScore positively correlated with the ratio of immune or stromal, and it referred to the higher the respective score and the larger the ratio of the corresponding component in TME. ESTIMATEScore was the sum of both, denoting the integrated proportion of both components in TME.

TIMER (Tumor Immune Estimation Resource) database (https://cistrome.shinyapps.io/timer/) was used to explore of the relevance between signature genes expression level and cancer cell associated fibroblast via EPIC algorithm (Li et al. 2017).

### Drug susceptibility analysis

The half maximal inhibitory concentration (IC50) is an important indicator to evaluate the efficacy of a drug or the response of a sample to treatment, and a lower IC50 indicates a higher anti-tumor ability (Gomaa 2017). To assess the chemotherapeutic sensitivity of the prognostic model, the prediction process was conducted using the R package “pRRophetic” (version 0.5) and the IC50 value estimate of chemotherapeutic drugs was estimated by ridge regression.

### The human protein atlas

The protein expression level of these proteins in tumors compared to normal tissue was obtained from the Human Protein Atlas database (https://www.proteinatlas.org/).

### Single cell communication analysis

The CellChat (version 1.6.1) R package was used for analysis cell communication. Identifying prognosis-associated subpopulations among single-cell and bulk sequencing data by Scissor R packages (version 2.1.0).

### Cell culture

Human ductal cell line (hTERT-HPNE) and pancreatic cancer cell lines, HPDEC, MIA PaCa-2, Capan-1, PANC-1 and CFPAC-1, were bought from ATCC. All cell lines were authentic by short tandem repeats profile. The HPDEC, MIA PaCa-2, Capan-1 (DMEM, Gibco, USA) and PANC-1 and CFPAC-1 (RPMI 1640, Gibco) were cultured in cell culture dishes (NEST Biotechnology, China) in humidified incubator at 37 with 5% CO_2_.

### RNA extraction and RT-qPCR

Total RNA was extracted with Trizol reagent (Invitrogen, USA), and then reverse transcription was performed using the HiScript II Q RT SuperMix kit for qPCR (Vazyme, R223) according to the manufacturer’s instructions. qPCR performed using the ChamQ SYBR qPCR Master Mix kit (Vazyme, Q311) in accordance with the manufacturer’s instructions. All PCR primers, including their internal reference sequences, were designed using Primer 5 (Table S2). Subsequently, quantitative PCR was conducted using a real-time PCR machine (Roche, LightCycler®96). Each experiment was independently repeated at least three times. The specific primers used are detailed in Supplementary Table 2.

### Statistical analysis

All statistical analyses were performed by R software (version 4.1.3). RT-qPCR assays were performed in three replicates and repeated three times independently. For statistical methods, the independent *t*-test or Mann–Whitney U test were utilized to compare continuous data, while the chi-square test or *Fisher* exact test were deployed to compare categorical data. The Kruskal–Wallis test was used to compare three groups or above. The KM method and the corresponding log-rank test were performed to identify the prognostic value of marker genes. Additionally, Spearman correlation was used to assess the correlation between two continuous variables. Statistical significance was defined as ^*^P < 0.05, ^∗∗^*P* < 0.01 and ^∗∗∗^*P* < 0.001.

## Results

### Identification of DEGs based on scRNA-seq and GEO datasets, and establishing prognostic model

**Fig. 1:**
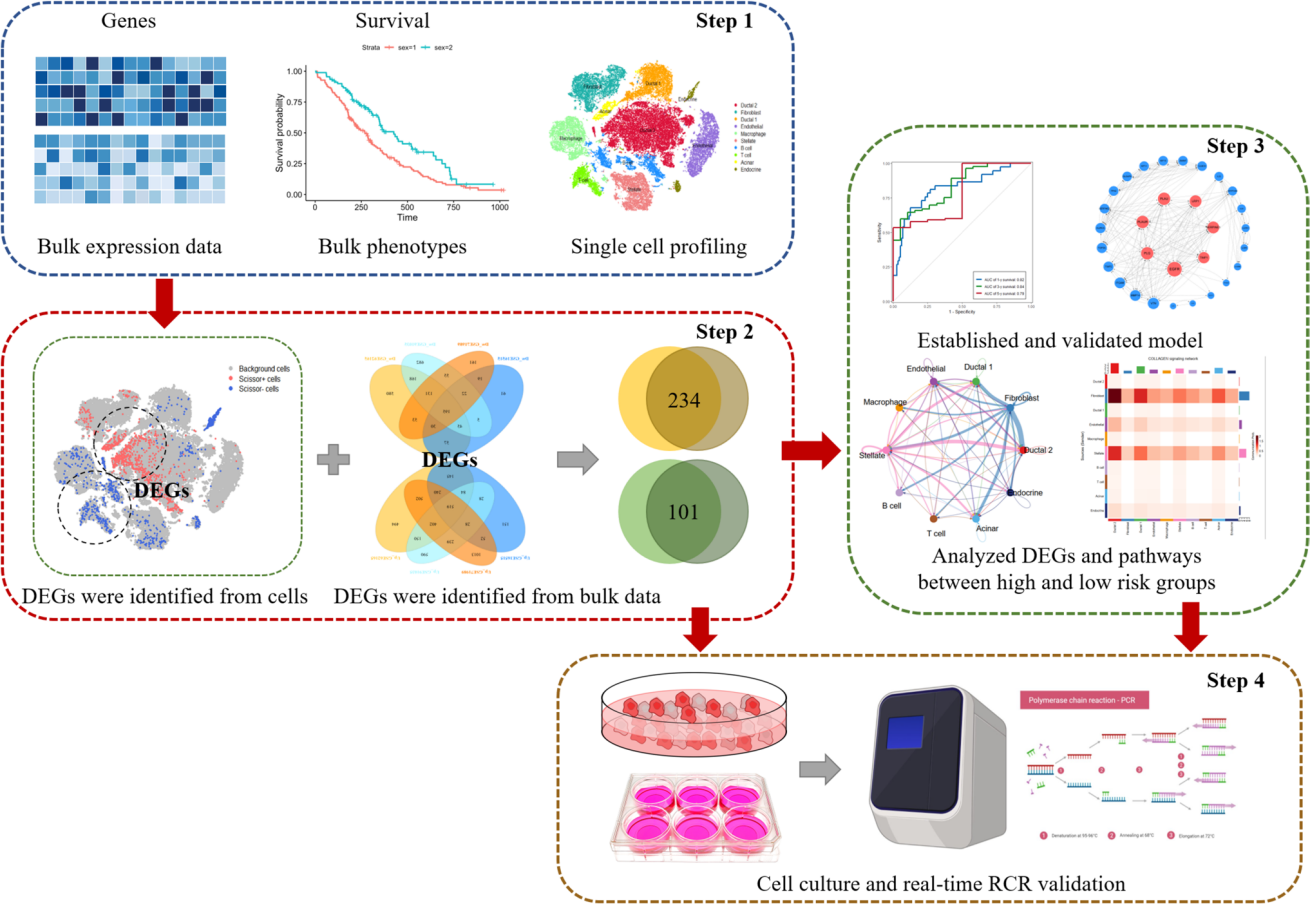
Graphical scheme describing the study design. Step1: The most and least correlated cell populations with prognostic phenotypes from 24 PDAC patients’ single-cell data were obtained by “Scissor” algorithm, and the DEGs between the two groups were obtained by the “FindMarkers” algorithm. Step2: The differential genes between PDAC and normal pancreas in four GEO datasets were analyzed, and then the common DEGs were identified by aggregate ranks. Step3: The key genes of the multivariate cox model were constructed based on TCGA data, and the internal and external data were used to diagnose the model. The correlation of modeled gene expression was analyzed to identify key genes. The model was used to divide the patients in different datasets into high and low risk groups, and the gene mutation landscape and immune infiltration were compared between the groups. The expression of modeling genes in cells was analyzed in the single-cell data to identify the pathways most relevant to prognosis. Step4: The expression of prognostic related genes in control cells and cells with different malignant degrees was detected, and the expression of pathway-related genes in different cells was verified

**Fig. 2:**
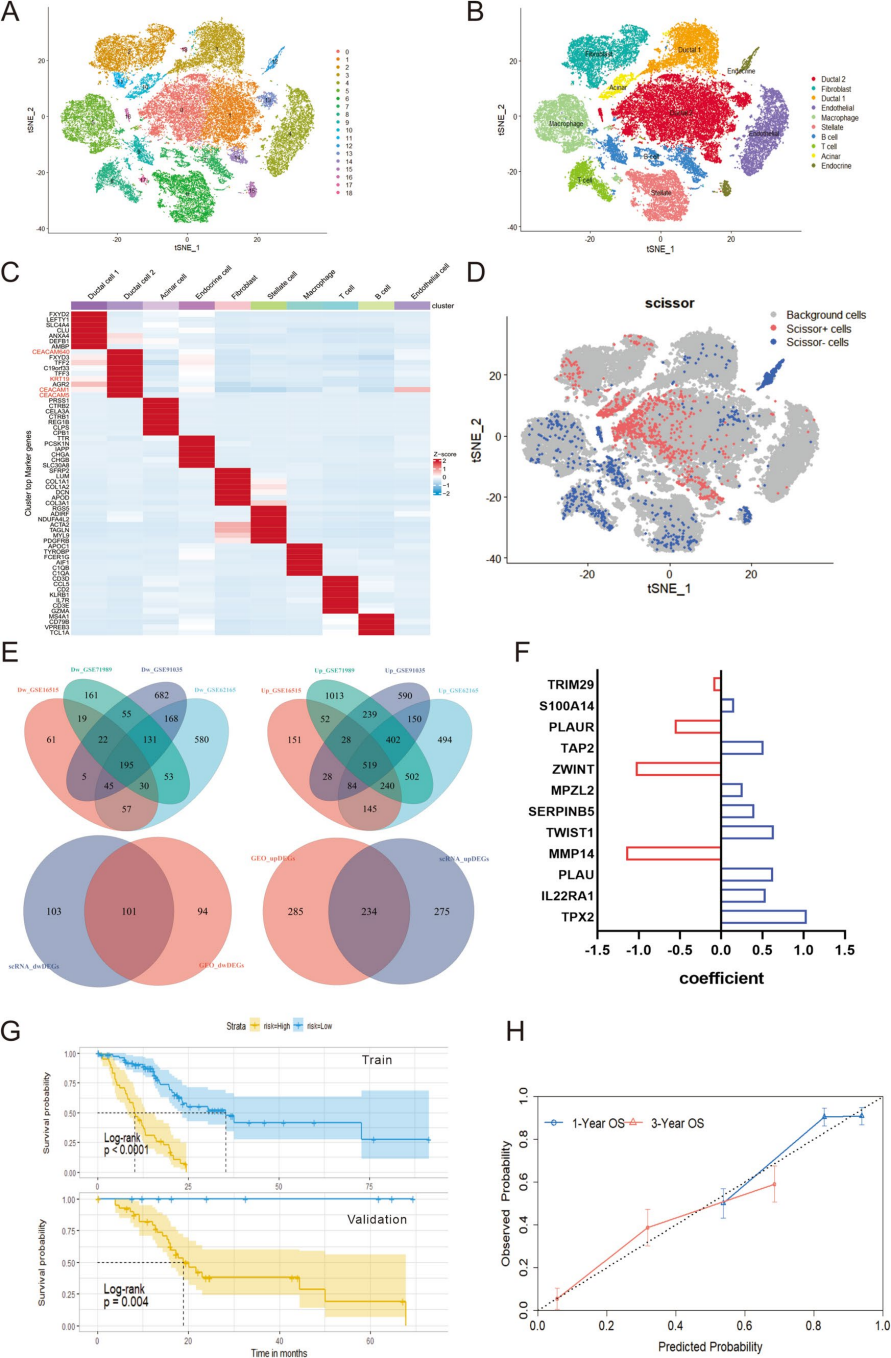
Screening of differential genes and construction of prognostic model. (**A**, **B**) The t-SNE plot showing the original cluster (**A**) and named cell subpopulations (**B**). **C** Heatmap showing the expression level of known cell-type-specific markers to demonstrate the identity of each cluster. **D** The t-SNE visualization of the Scissor selected cells. The red and blue dots are cells associated with the prognosis of tumor. **E** Venn diagram of the intersection of differential genes from the four GEO data and the intersection of differential genes derived from the scRNA-seq data and those derived from the GEO data. **F** Bar graph of coefficients from the multivariate cox regression model. **G** Survival curves of high-risk and low-risk groups in the training and testing sets. **H** Calibration curves of cox regression model for predicting one-year and three-year survival rates

The full-text analysis flow is shown in Fig. 1. To reveal the differences in gene expression profiles between tumor cells and normal ductal cells in PDAC, we downloaded single-cell transcriptome sequencing dataset from Genome Sequence Archive (GSA). A total of 24 PDAC (38 201 cells) specimens were included to construct gene-cell expression matrix. After cells filtering, normalization, principal component analysis (PCA) and *t*-distributed stochastic neighbor embedding (*t*-SNE) dimensionality reduction, 19 original clusters were identified (Fig. 2A). According to signature genes of each cell type reported previously (Chen et al. 2021b; Elyada et al. 2019; Peng et al. 2019), these clusters were classified into ten known cell types, including type 1 ductal, type 2 ductal, acinar, endocrine, endothelial, fibroblast, stellate, macrophage, T and B cells (Fig. 2B). We subsequently identified cluster-specific marker genes by conducting differential gene expression analysis to characterize the identity of each cell cluster (Fig. 2C, Supplementary Material, Fig. S1A). It was observed that type 2 ductal cells exhibited significantly higher expression of reported poor prognosis PDAC markers, such as *CEACAM1/5/640* and *KRT19*. In addition, we compared the composition of cells in 24 PDAC patients (Supplementary Material, Fig. S1B). The result showed that there were differences in the composition of tumor cells in different patients, while ductal cells and fibroblasts were the main cells, and lymphocytes accounted for a small proportion. To further integrate patient prognosis with single-cell data, we used the Scissor algorithm to identify the cell populations most relevant to prognosis at the cellular level (Sun et al. 2022). Based on the signs of the estimated regression coefficients, the cells with non-zero coefficients can be indicated as Scissor positive (Scissor+) cells and Scissor negative (Scissor-) cells, which are positively and negatively associated with the phenotype of interest, respectively, and differential genes between the two populations of cells are calculated using the “FindMarkers” algorithm (Fig. 2D). Four GEO datasets (GSE62165, GSE71989, GSE16515 and GSE91035) were normalized separately, and the differential genes between tumor and normal tissues were analyzed by “limma” R package, and the intersection of the differential genes of the four datasets was taken. Finally, 101 down-regulated genes and 234 up-regulated genes were obtained by intersection of the differential genes obtained in the scRNA-seq data and the differential genes in the GEO datasets (Fig. 2E). The PDAC cohort in TCGA was randomly assigned to the training set and the validation set according to 7:3. We performed Unicox regression analysis on 335 differentially expressed genes (DEGs) found in training set, found 145 DEGs significantly related to prognosis (*P* < 0.01). Then, Lasso regression (Supplementary Material, Fig. S1C–D) and multivariate cox regression models were used to further screen the prognostic genes. Ultimately, 12 genes, including *TRIM29*, *S100A14*, *PLAUR*, *TAP2*, *ZWINT*, *MPZL2*, *SERPINB5*, *TWIST1*, *MMP14*, *PLAU*, *IL22RA1* and *TPX2*, were confirmed as independent prognostic DEGs, and the coefficients of their constructed risk score of the multivariate cox model are shown in the Fig. 2F. All 12 gene met the proportional hazards assumption using Schoenfeld residuals (Supplementary Material, Fig. S1E). The ROC curve analysis showed that the area under the curve of the model in the training set and validation set was 0.76 and 0.67, respectively (Supplementary Material, Fig. S1F). The coefficients of the individual prognostic genes in the model are shown in Fig. 2F, and the survival curve showed that the OS of patients in the high-risk group was poor in the training set and validation set (Fig. 2G). The calibration curves of the model in the validation dataset, and it showed good correlation between nomogram-predicted OS and actual OS, indicating the accuracy of the prognostic model (Fig. 2H).

### Validation of diagnostic model

**Fig. 3:**
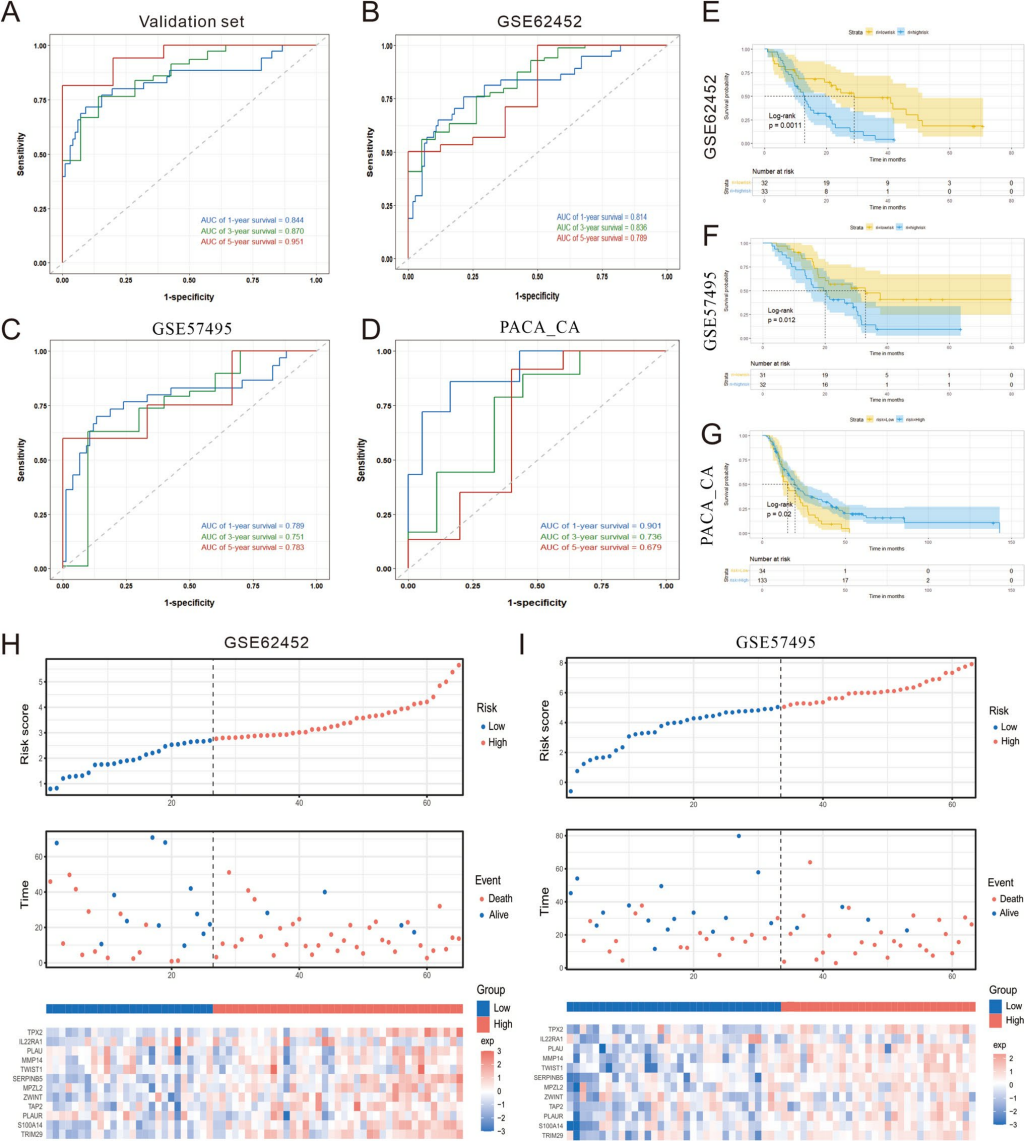
Internal and external datasets were used to validate the prognostic model. **A**–**D** 1-year, 3-year, and 5-year time-ROC curves evaluate the risk stratification ability and predictive ability of the constructed risk model in the TCGA validation data, GSE62452, GSE57495 and ICGC PADA_CA cohorts. **E**–**G** The Kaplan–Meier survival curves show the clinical relevance of the PDAC signature on three independent datasets (GSE62452, GSE57495 and ICGC PADA_CA). Tick marks indicate censoring events. The statistical *p*-values were determined by the two-tailed log-rank sum test. **H**–**I** Risk plots to illustrate the survival status of each sample and signature genes expression heatmaps in the GSE62452 and GSE57495 cohorts

Prognostic models were validated on internal and external data sets. Time-dependent receiver operating characteristic curve (ROC) was used to evaluate the accuracy of predicting 1-year, 3-year, and 5-year OSs. The area under curve (AUC) values were 0.844, 0.870 and 0.961 in the internal validation set (Fig. 3A). To further evaluate the accuracy and reliability of the prognostic model, we retrieved gene expression matrix and clinical follow-up data of PDAC from GSE62452 GSE57495, and ICGC (PACA_CA) as an external validation set. Their 1-/3-/5-year AUC values were 0.814/0.836/0.789, 0.789/0.751/0.783 and 0.901/0.736/0.679 (Fig. 3B-D). Subjects in the high-risk group had significantly shorter OS than those in the low-risk group based on four prognosis-related signatures in all external validation sets for GSE62452 (*P* = 0.0011), GSE57495 (*P* = 0.012), and PACA_CA (*P* = 0.02) (Fig. 3E-G). The risk plots and signature genes expression heatmaps were generated to show detailed survival outcomes of each patient in the external validation cohorts (Fig. 3H–I, Supplementary Material, Fig. S2A).

### Clinical relevance, nomogram and mutation landscape between high- and low-risk groups

**Fig. 4:**
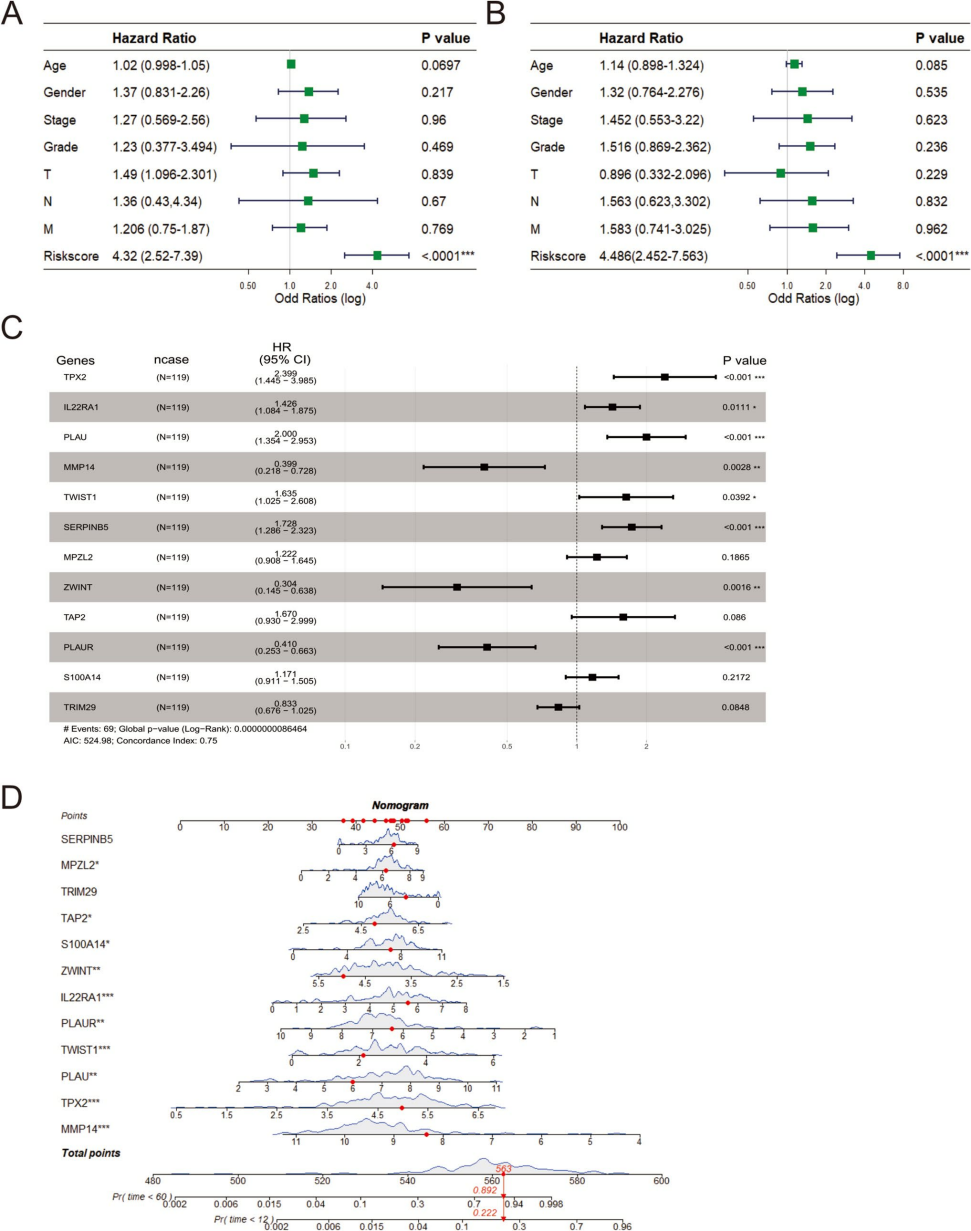
Unicox and multicox analysis, and nomogram established. **A** The forest plots show the hazard ratios and 95% confidence intervals for the risk-score and additional clinical features according to the Unicox model. Squares represent the hazard ratios and the horizontal bars extend from the lower limits to the upper limits of the 95% confidence intervals of the estimates of the hazard ratios. The statistical *p*-values were determined by the two-tailed Wald test. **B** The forest plots show the hazard ratios and 95% confidence intervals for the risk-score and additional clinical features according to the multivariable Cox model. **C** The forest plots show the hazard ratios and 95% confidence intervals for the multivariable Cox model. **D** The prognosis nomogram was drawn to predict 1-year and 5-year OSs for PDAC. Red point on the diagram to illustrate patients through calculation of the nomogram survival probability and the probability of less than 1 year and 5 years

We performed single factor and multi-factor Cox analyses to determine whether the risk score could be an independent prognostic factor for PDAC patients compared with other common clinicopathological parameters. We observed that the risk score could serve as an independent prognostic factor for these individuals (Fig. 4A). The Unicox results showed that risk score (HR: 4.486, 95% CI: 2.452–7.563, *P* < 0.0001) were significantly correlated to the OS of subjects. Multivariable Cox regression analysis of the model showed that except *S100A14*, *TAP2*, *MPZL2* and *TRIM29*, other genes were significantly correlated with survival time (Fig. 4B-C). Next, we investigated the relationship between the risk score and clinicopathological features, suggesting that TNM stage were not significantly associated with the risk score (Supplementary Material, Fig. S2B). Furthermore, we established the easy-to-use and clinically adaptable prognostic nomogram. The subject with higher total points was associated with worse 1-year and 5-year OSs (Fig. 4D). Afterward, waterfall plots of the PDAC cohort as a whole and the high- and low-risk PDAC groups were generated to explore the detailed mutational profiles between the two groups (Supplementary Material, Fig. S2C-D). We found that *KRAS*, *TP53*, and *CDKN2A* were the most frequently mutated genes in the high- and low-risk groups, and the gene mutation frequency in the high-risk group was higher than that in the low-risk group. Fisher test found that *KRAS* and *TP53* were significantly mutated genes between the two groups (Supplementary Material, Fig. 2E).

### Differential gene expression analysis and chemotherapy drug sensitivity between high-risk group and low-risk group

**Fig. 5:**
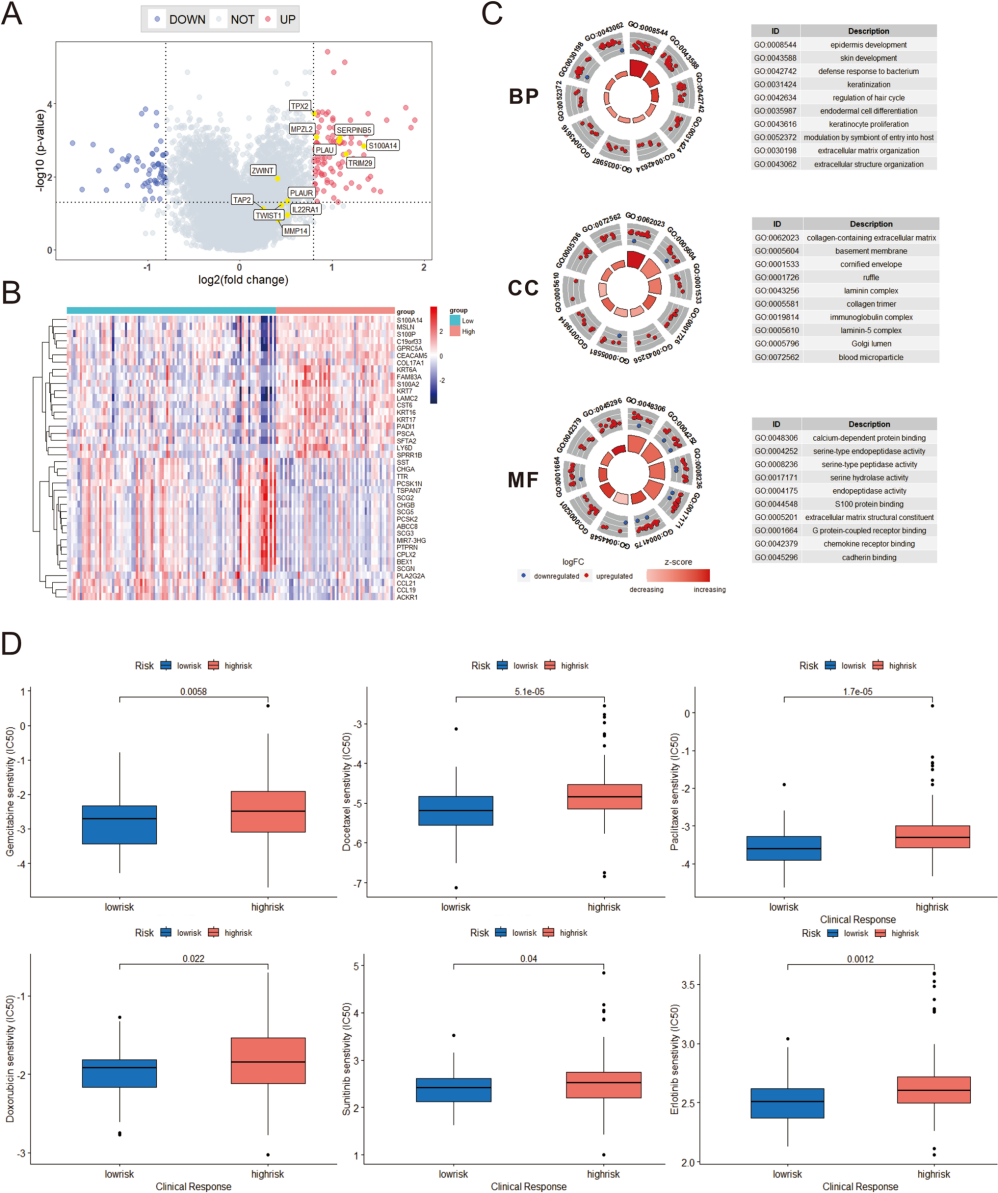
Differential genes, related pathways and drug sensitivity between high and low risk groups were analyzed. **A** The volcano plot of differential gene expressions in high-risk versus low-risk. The two vertical dashed lines represent ± ln (2.17) fold-changes in gene expression, and the horizontal dashed line denotes FDR cutoff 0.05. The FDR was the adjusted *p*-value calculated by the two-tailed Wilcoxon rank-sum test. **B** Heatmap of high- and low-risk group differences in genetic analysis. **C** BP, CC and MF analysis of differentially expressed genes between high- and low-risk groups. **D** Drug sensitivity analysis of high- and low-risk groups in TCGA cohort. Estimated IC50 for Docetaxel, Gemcitabine, Paclitaxel, Doxorubicin, Sunitinib, and Erlotinib

The “limma” algorithm was employed to calculate the differential expression of genes between the high-risk and low-risk groups in the TCGA dataset. Additionally, the expression levels of genes associated with prognosis were examined (Fig. 5A-B). The results showed that not all modeling genes were differentially expressed between high- and low-risk groups. Furthermore, GO and KEGG enrichment analysis of 167 gene signatures was performed by Cluster Profiler packages in R project (Fig. 5C, Supplementary Material, Fig. S3A). PI3K-Akt signaling pathway, collagen-containing extracellular matrix, and calcium-dependent protein binding were significantly enriched. Chemotherapy drugs, such as Gemcitabine (Qi et al. 2023) and Docetaxel (Fan et al. 2020), have remained the mainstay for the treatment of PDAC. Poor prognosis has been associated with chemoresistance. Therefore, we further predicted the chemotherapy response of the two risk subgroups to common chemotherapy drugs. As shown in Fig. 5D, a significantly higher estimated IC50s for six chemotherapy drugs (Docetaxel, Gemcitabine, Paclitaxel, Doxorubicin, Sunitinib and Erlotinib) of high-risk group when compared with low-risk group, which indicate that low-risk patients can benefit from the chemotherapy agents. In addition, we also observed the sensitivity of patients to chemotherapy drugs in the GSE62452 cohort (Supplementary Material, Fig. S3B), and found that the low-risk group was more sensitive to Gemcitabine, Paclitaxel, Doxorubicin, and Sunitinib, while the high-risk group was more sensitive to Docetaxel and Erlotinib.

### The immune microenvironment and immune checkpoints of high- and low risk groups

**Fig. 6:**
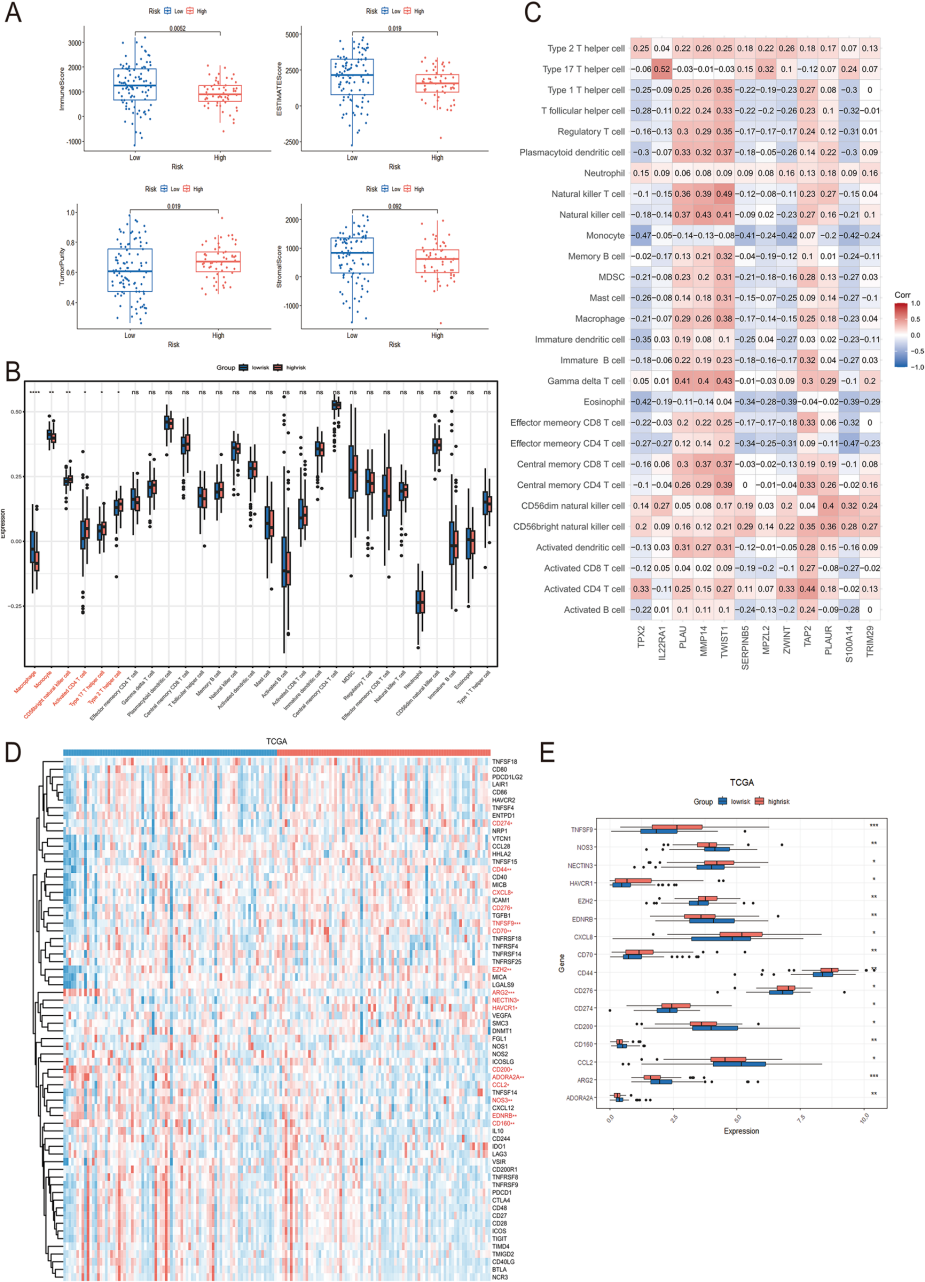
Immunity landscape and check point inhibitors genes expression pattern of high- and low-risk groups. **A** The ESTIMATE algorithm was used to calculate the immune score, ESTIMATE score, tumor purity score and stroma score between high- and low-risk groups. **B** Immune cells infiltration levels in the low- and high-risk groups estimated by ssGSEA. **C** Spearman analysis plots of correlation between signature gene expression and immune cells infiltration levels. **D** Heatmap of Cancer-Immunity Cycle-related genes and immune checkpoint gene expression patterns in the high- and low-risk groups. **E** Boxplot of representative immune-related genes expression between high- and low-risk groups

We analyzed the differences of tumor microenvironment in the high- or low-risk groups. The tumor purity, immune score, stromal score and ESTIMATE score were calculated by ESTIMATE algorithm, which calculated based on single-sample gene set enrichment analysis (Fig. 6A). Except for stroma score, significant differences were found in immune score, tumor purity and ESTIMATE score between the high and low risk groups. Based on the results of previous studies on pan-cancer immune genes (Charoentong et al. 2017), ssGSEA was employed to analyze the abundance of different immune cells in both the high-risk and low-risk groups in TCGA (Fig. 6B) and the GEO cohort (Supplementary Material, Fig. S4A-B). The results revealed significant differences in the abundance of macrophages, monocytes, CD56^+^ natural killer cells, activated CD4 T cells, Th17 cells, and Th2 cells between the high-risk and low-risk groups within the TCGA cohort. Additionally, macrophages, monocytes, activated CD4 T cells, and Th2 cells exhibited significant differences in both GEO cohorts (GSE62452 and GSE57495). Furthermore, dendritic cells (both immature and activated) were identified in the analysis of both cohorts. Lastly, we also investigated the association between the signature genes and 28 tumor-infiltrating lymphocytes of TCGA dataset (Fig. 6C). Spearman’s correlation analysis revealed that *PLAU*, *MMP14*, *TWIST1*, and *TAP2* was positively correlated with the expression level of most tumor-infiltrating lymphocytes, while *TPX2*, *SERPINB5*, *MPZL2*, and *S100A14* was negatively correlated with monocytes, plasmacytoid dendritic cells, type 1 T helper cells, regulatory T cells, and eosinophils. Anti-cancer immune response can be conceptualized as a series of stepwise events referred to as the Cancer-Immunity Cycle (Xu et al. 2018). At the same time, the expression level of immune checkpoint related molecules is closely related to the outcome of immunotherapy. Therefore, we summarized 74 pan-cancer immune checkpoint genes and molecules related to tumor immune cycle found in previous studies (Park et al. 2021), and visualized the expression levels of these molecules in high- and low-risk groups of TCGA, GEO and ICGC cohorts (Fig. 6D-E, Supplementary Material, Fig. S4D-E). Compared with low-risk group, *ARG2*, *NECTIN3*, *HAVCR1*, *CD274*, *CD160*, *NOS3*, *EDNRB*, *CCL2*, and *CXCL8* were significantly higher expression in high-risk group. As for the expression level of common immune checkpoint related genes of TCGA, GEO and ICGC cohorts, the results revealed that a higher risk score was significantly associated with up-regulation of *CD274* (*PD-L1*), *CD44*, *CD70*, *CD276*, and *TNFSF9* and down-regulation of *CD160*, *ADORA2A*, and *CD200* in TCGA dataset.

Taken together, the above results showed that there were differences in tumor purity and immunity level between the high- and low-risk groups, and there were significant differences in macrophages, monocytes and CD4^+^ activated T cells between the two groups. At the same time, the modeling genes were correlated with the expression levels of immune cells, suggesting that the survival difference between the high- and low-risk groups may be induced by the difference in the immune level of myeloid cells within tumor.

### Expression analysis, PPI network identification and cytological experiment verification of prognostic related molecules

**Fig. 7:**
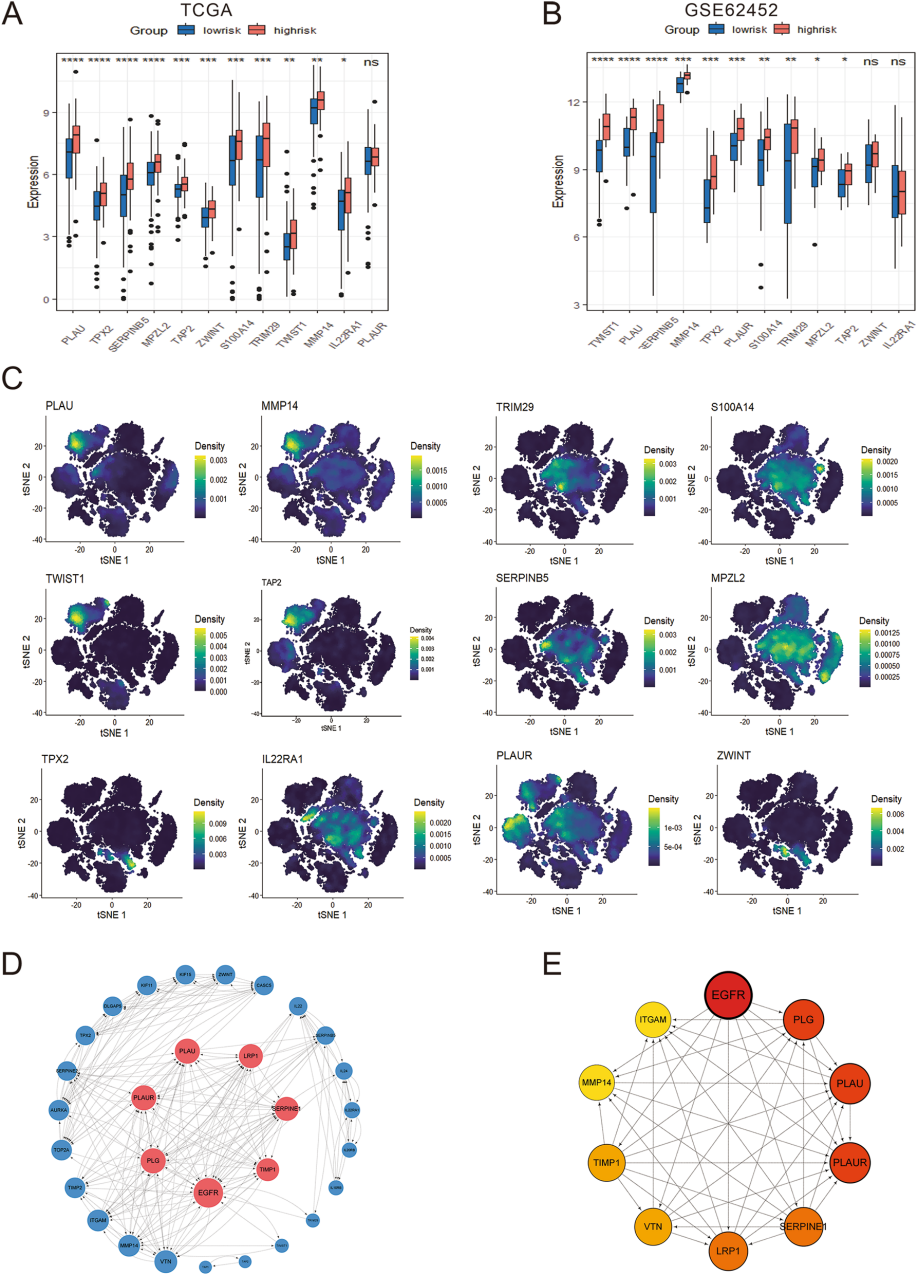
Expression analysis and PPI network identification. **A**–**B** The boxplot shows the expression levels of signature genes in TCGA and GSE62452 cohorts. **C** Density plot of expression distribution of 12 prognostic related genes in single cell data. **D** According to the contribution degree to build the PPI network nodes. Each node represents a protein, while each edge represents the interaction between two proteins, and the greater the contribution degree, the greater the node. **E** Identification of hub genes network by cytoHubba app in Cytoscape

**Fig. 8:**
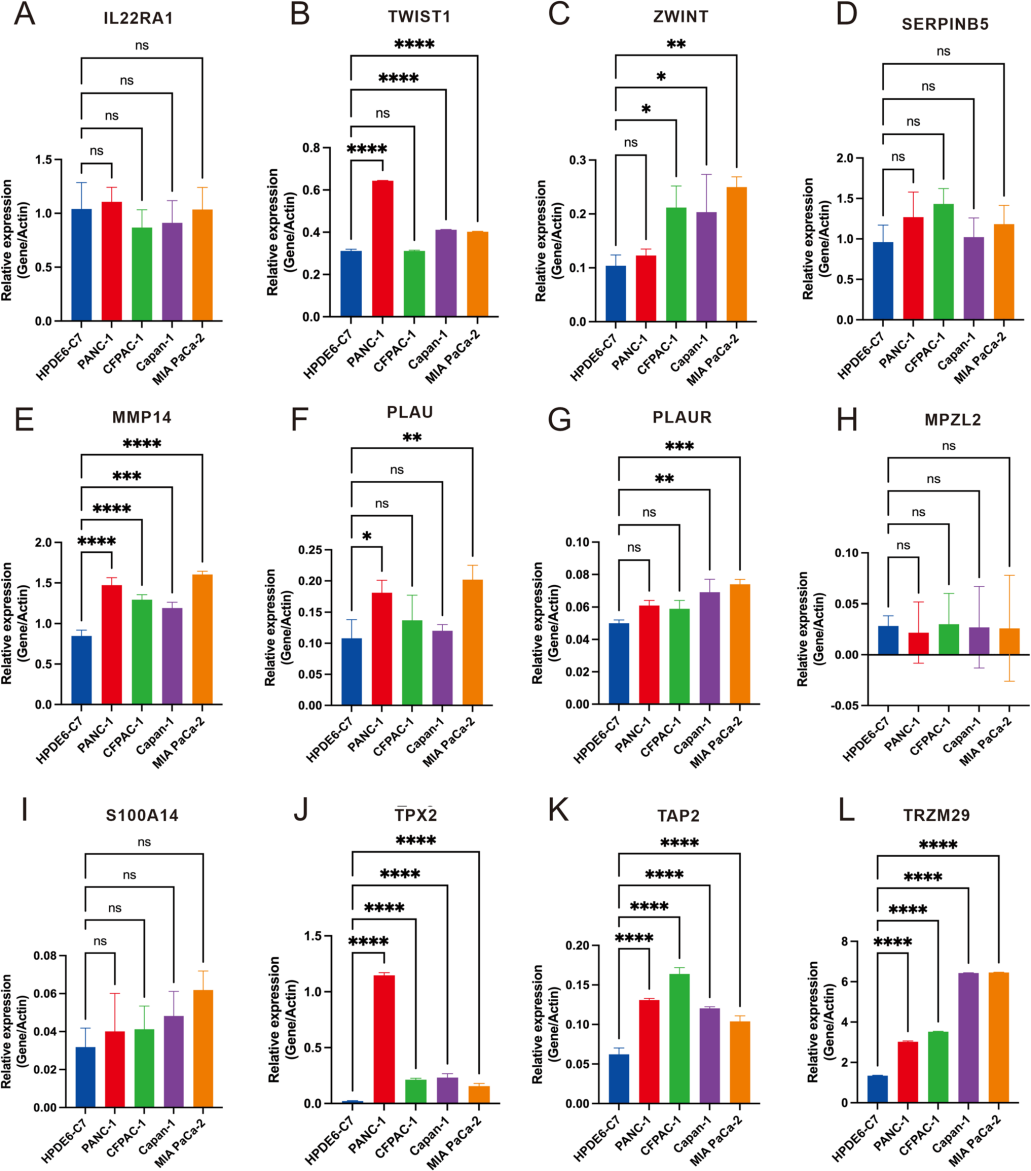
Cytological experiment verification of prognostic related molecules. **A–L** Real-time PCR was used to detect *IL22RA1* (**A**), *TWIST1* (**B**), *ZWINT* (**C**), *SERPINB5* (**D**), *MMP14* (**E**), *PLAU* (**F**), *PLAUR* (**G**), *MPZL2* (**H**), *S100A14* (**I**), *TPX2* (**J**), *TAP2* (**K**), and *TRIM29* (**L**) expression in HPDE6-C7 (Normal epithelial cells), PANC-1 (Metastatic type cell), CFPAC-1 (Metastatic type cell), Capan-1 (Carcinoma in situ) and MIA PaCa-2 (Carcinoma in situ) cells

In order to further clarify the expression of the 12 prognostic genes, we compared the expression of genes in TCGA and GEO datasets (GSE62452, GSE57495), and found that most genes were significantly different between the two groups (Fig. 7A-B, Supplementary Material, Fig. S5A). The distribution of prognostic genes in cells was further verified in single-cell data, and it was found that these genes were mainly highly expressed in ductal type 2 cells, fibroblasts, acinar cells and macrophages (Fig. 7C). The list of 12 signature genes was uploaded to STRING online database and then the PPI network was visualized by Cytoscape (Fig. 7D). The nine genes with the highest contribution degree of nodes in the network were selected as the hub genes of the whole pathway for protein interaction pathway analysis (Fig. 7E). As can be seen from the plot, *EGFR*, *PLG*, *PLAU* and *PLAUR* have the largest contribution in the protein–protein interaction network, which may be the key regulators in determining the prognosis of patients, which suggest that the differences in prognosis may be due to these kinds of cells. Subsequently, we determined the expression levels of prognostic related genes by real-time PCR in healthy control cells and pancreatic cancer cells with different degrees of malignance (Fig. 8A-L). The results showed that except for *SERPINB5*, *S100A14*, *IL22RA1* and *MPZL2*, the expression levels of other genes were increased along with the increase of cell malignancy. The expression of 10 signature genes was also validated in the tumor samples and normal samples in HPA database (Supplementary Material, Fig. S5B). The result showed that except for *TWIST1* and *IL22RA1*, other ten protein expression levels were significantly increased in PDAC tissues compared to their normal tissues.

### Communication analysis of cell subsets

**Fig. 9:**
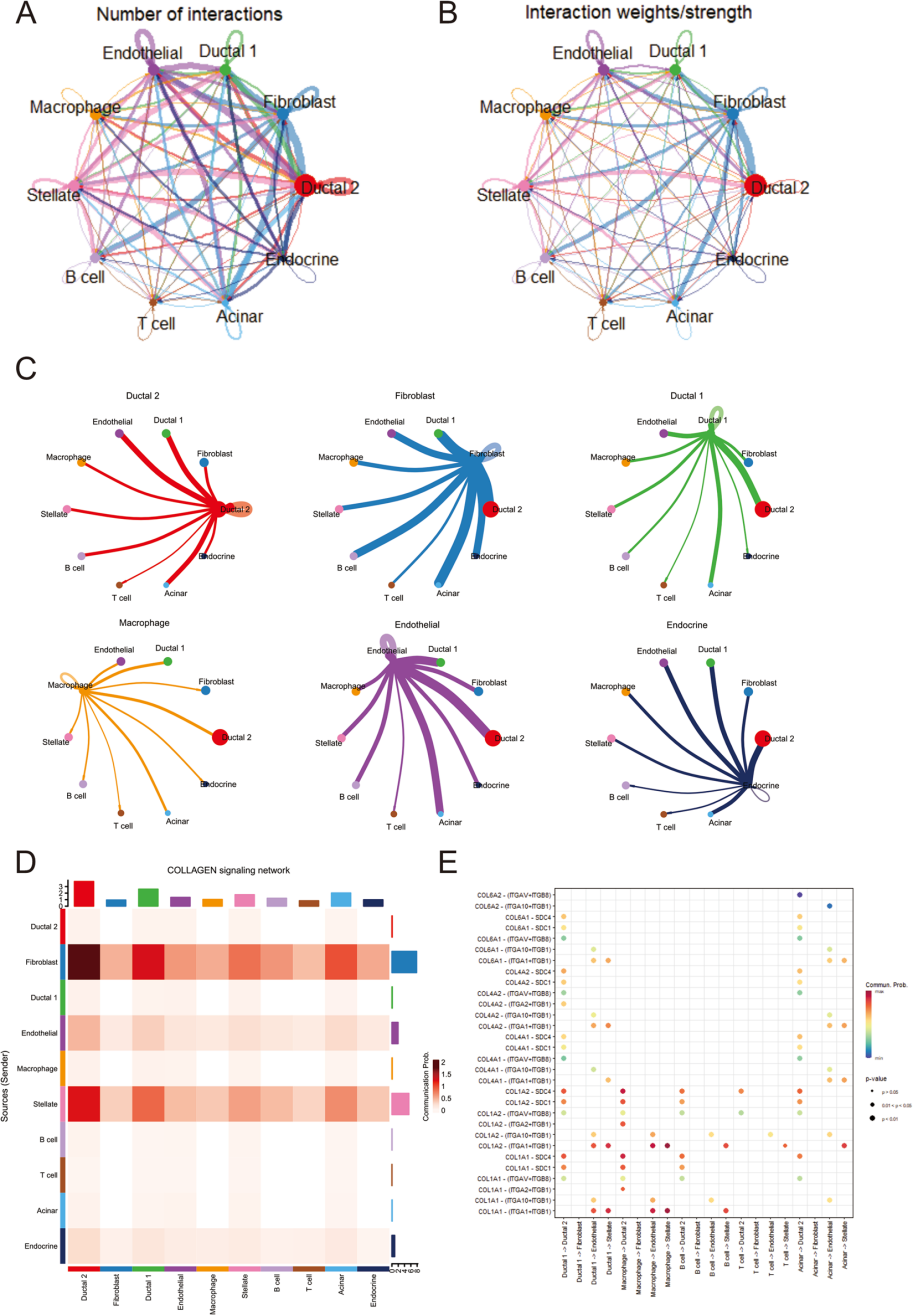
Cell subsets communicate with their receptors. **A** Network diagram of the number of cell-to-cell interactions. **B** Network diagram of the strength of cell-to-cell interactions. **C** Plot of the intensity of interactions between a single cell and other cells. **D** Heatmap of collagen pathway interaction strength between cells. The vertical axis is the cell that emits the signal, the horizontal axis is the cell that receives the signal, and the accumulation of the intensity of the vertical and horizontal axes is shown on the upper side. **E** Bubble plots of signal intensities of collagen pathway ligands and receptors between cell subsets

To further investigate the interactions between cell subsets that are associated with prognosis, cell communication of 10-cell subsets was analyzed by Cellchart (Fig. 9A–C). From Fig. 9A, it can be seen that the interaction of these 10 subsets changes in the number and intensity of ligand‒receptor interactions, and strong cell communication exists between fibroblasts, acinar, type 2 ductal cells, and epithelial cells. When examining individual signaling pathways or ligand-receptor mediated cell interactions, the collagen pathway was found to be highly interactive between fibroblasts and type 2 ductal cells, type 1 ductal cells and acinar cells (Fig. 9D, Supplementary Material, Fig. S6A). Further analysis of the ligand-receptor interactions between cells and their contributions to the collagen signaling pathway showed that macrophages had strong interactions with ductal cells and epithelial cells, and CD44 molecules and COL1A2 molecules contributed the most to the pathway (Fig. 9E, Supplementary Material, Fig. S6B).

### Cytological validation of cell interaction pathways

**Fig. 10:**
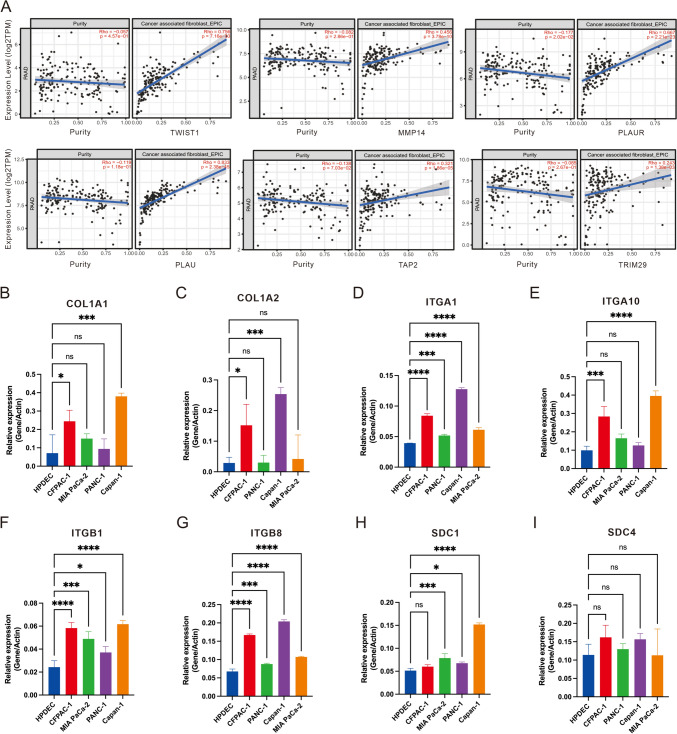
Correlation between the level of fibroblast infiltration and the expression of prognostic molecules and cytologic verification of the expression levels of key molecules in the cellular collagen formation pathway. **A** 6 signature genes correlate with tumor purity and is significantly positively associated with fibroblast infiltrates using the TIMER2.0 database. **B–I** Bar plot to verify the expression levels of crucial molecules in the collagen formation pathway, including *COL1A1*, *COL1A2*, *ITGA1*, *ITGA10*, *ITGB1*, *ITGB8*, *SCD1*, *SCD4*, in normal pancreatic ductal cells and cells with different degrees of malignancy

Since the collagen formation pathway was found to be closely related to fibroblasts in the single-cell analysis results, we further verified the correlation between the expression levels of prognostic related molecules and fibroblasts in the TIMER2.0 database. (Fig. 10A). The results showed that the expression of 6 genes were significantly positively correlated with the infiltration of fibroblasts in the tumor. We further examined the expression of crucial molecules in the collagen formation pathway in pancreatic cancer cell lines (Fig. 10B-I). The results showed that *ITGA1*, *ITGB1*, *ITGB8* and *SDC1* were highly expressed in tumor cells when compared with control group. These results suggested that the prognosis of patients with PDAC was likely to be related to the interaction between fibroblasts and ductal cells, and the interaction may be mainly mediated by collagen formation.

## Discussion

Pancreatic ductal adenocarcinoma (PDAC) is a highly malignant and aggressive solid tumor, which is characterized by atypical symptoms, hidden location, rapid disease progression and poor prognosis (McGuigan et al. 2018). The 5-year survival rate of PDAC is only 8% (Hezel et al. 2006). Although surgical resection and postoperative administration of gemcitabine or other biological targeted therapy remain the main treatment options for PDAC, only 10% to 15% of newly diagnosed patients are eligible, and most patients who are resistant to gemcitabine will eventually die of metastasis due to recurrence and chemotherapy failure (Chen et al. 2021a, 2022; Yuan et al. 2021). Therefore, the discovery of novel PDAC biomarkers is essential for prognostic prediction and development of novel drug targets.

In pancreatic cancer tissue, malignant tumor cells account for only a small fraction of the tumor component; the remainder is mostly composed of extracellular matrix, pancreatic stellate cells, and fibroblast proliferation (Bhatia et al. 2022; Girish et al. 2022; Wood et al. 2022). In addition, pancreatic cancer has an extensive immunosuppressive microenvironment that promotes cancer cell proliferation by directly suppressing antitumor immunity or evading immune surveillance (Mills et al. 2022). Therefore, prognostic modeling of patients solely from bulk RNA sequencing data may not achieve the desired results. The popularization of single-cell detection technology has promoted the study of intratumorally heterogeneity. RNA expression profiles in different cell types can be obtained by single-cell assays and cell reference genes (Peng et al. 2019). However, the current single-cell sequencing cohorts are generally small, which cannot meet the sample size requirements for establishing prognostic prediction models. Combining the results of bulk RNA-seq cohorts with single-cell sequencing may address this issue (Liao et al. 2021).

In this study, we first identified the most and least prognostic related cell subsets from single-cell data by the Scissor algorithm to obtain differentially expressed genes between the two groups of cells. We also considered that expanding the testing cohort may provide a better extrapolation model, so we selected 4 high quality GEO cohorts and obtained the differentially expressed genes between their groups. Finally, the differentially expressed genes obtained from the single-cell data and the GEO cohort were intersected to obtain 335 differentially expressed genes. Then Unicox regression analysis and Lasso regression analysis was used to analyze the number of compressed variables, and finally a prognostic prediction model based on 12 prognostic related genes was established. Through the time-ROC curve and calibration curve test, it was found that the model could effectively distinguish patients with different prognostic outcomes. Three independent data sets were used to verify the effect of the model, and it was found that the model had certain extrapolation. To date, at least 11 out of 12 selected genes have been identified as prognostic factors for PDAC in other studies (Atay 2020; Cantero et al. 1997; Chen et al. 2021c; Guenther et al. 2023; Hosen et al. 2022; Slapak et al. 2020; Sun et al. 2014; Wang et al. 2020; Zhang and Yang 2022; Zhu et al. 2021). The importance of addressing the use of Cox and Lasso for marker selection should be emphasized.

Then the population was divided into high- and low-risk groups by the model, and the differential genes, pathway enrichment and immune status differences between the two groups were explored. It was found that there were significant differences in collagen formation pathway, the infiltration proportion of macrophages and monocytes between the two groups. Further analysis of the distribution of prognostic genes in cell subsets by single-cell data showed that 12 genes were mainly enriched in ductal type 2 cells (highly associated with malignant differentiation), cancer-associated fibroblasts (CAF) and macrophages. The results of cytological experiments showed that prognostic related molecules were generally highly expressed in metastatic cells, so it was highly suspected that the source of the prognosis difference between the high and low risk groups was related to CAF and collagen formation pathway. Analysis of intercellular communication by single-cell sequencing data revealed a strong interaction between CAF and ductal type 2 cells, in which the collagen formation pathway was the most powerful. By cell assay, it was found that the expression of key genes in the highly malignant collagen formation pathway was increased.

In previous studies, CAF is an important component of the tumor microenvironment, and the aggregation and activation of CAF can reshape the extracellular matrix (ECM) of the tumor microenvironment, leading to its precipitation and changes in the proliferation and infiltration of tumor tissue, angiogenesis and immune response, and eventually lead to the proliferation and fibrosis of PDAC connective tissue. CAF plays an important role in the progression of PDAC. In the present study, *PLAU*, *MMP14*, *TWIST1*, and *TAP2* genes among the 12 prognostic related molecules were enriched in CAF, with PLAU and MMP14 acting as hub genes in protein–protein interactions. In previous studies, *Fang* et al. (Fang et al. 2021) found that *PLAU* promotes the proliferation of esophageal squamous cell carcinoma (ESCC) cells through the MAPK pathway, and also promotes metastasis by up-regulating slug and MMP9. Additionally, *PLAU* can promote the transformation of tumor fibroblasts into inflammatory CAF. Furthermore, IL8 secreted by CAF can further promote the expression of *PLAU* in tumor cells, thereby promoting the development of ESCC. Furthermore, in 3D cell culture systems constructed by Rizwan et al., it was discovered that the expression of *PLAU* and other genes was upregulated when tumor cells were co-cultured with CAF. This finding could potentially be used as a target for drug therapy (Ali et al. 2021). Compared to *PLAU*, the role of *MMP14* in CAFs has been more extensively studied. *Noda* et al. discovered that *MMP14* expression in tumor nests and CAFs, as well as its overexpression at the tumor-stromal interface, significantly correlated with the presence of extranodal extension (ENE) in a retrospective cohort study (Noda et al. 2023). In a retrospective analysis conducted by *Makutani* et al., tumor samples from 86 patients with stage III colorectal cancer were examined. The study revealed that *MMP14* was highly expressed in intratumoral CAF, and patients with tumors showing high expression of MMP14 in CAF had a poor prognosis (Makutani et al. 2022). Although current studies have uncovered the mechanism of action of these two molecules in a variety of CAF, their mode of action in CAFs of PDAC has not been fully reported. Meanwhile, immune infiltration analysis revealed significant differences in macrophages and monocytes between different risk groups. Transcriptome sequencing data, mutation data, and corresponding clinical information of PAAD were obtained from the TCGA database. According to previous studies, CAF have been shown to inhibit the differentiation and infiltration of macrophages, thereby exerting an immunosuppressive effect (Tang et al. 2022). In line with the original authors of the single-cell data, we also identified type 2 ductal cells that exhibited high expression of poor prognostic molecules such as *CEACAM1/5/640* and *KRT19*, suggesting their potential malignancy. Intercellular interaction analysis revealed a strong interaction between type 2 ductal cells and fibroblasts, with collagen formation being the predominant pathway of interaction. Therefore, we strongly suspect that the disparity in prognosis may be attributed to the influence of malignant ductal cells on the differentiation of CAF cells through the collagen formation pathway. Subsequently, CAF may further interfere with angiogenesis and macrophage differentiation, thereby contributing to the observed differences in prognosis.

This study has a few limitations. Firstly, the performance of the established model in ICGC data is not specific and, compared to other models, the model that uses 12 genes as features may be slightly redundant. Although multicollinearity between the modeled molecules was not detected, the key molecules can be further streamlined in future studies. Secondly, in the pathway verification, only simple experimental observations were conducted on the key molecules, and the deeper mechanistic pathways were not explored. Future research can delve into the relevant molecular mechanisms.

In summary, our findings have successfully established and validated a prognostic prediction model for PDAC from multiple perspectives. This study provides a valuable resource for understanding intratumor heterogeneity, elucidates the connection between intra-tumoral CAF cells and PDAC prognosis, and identifies potential biomarkers for targeted therapy and immunotherapy, thereby offering promising avenues for anti-tumor treatments.

## Supplementary Information

Below is the link to the electronic supplementary material.

Supplementary file1 (DOCX 1173 KB)

Supplementary file2 (DOCX 1060 KB)

Supplementary file3 (DOCX 393 KB)

Supplementary file4 (DOCX 2721 KB)

Supplementary file5 (DOCX 2186 KB)

Supplementary file6 (DOCX 560 KB)

Supplementary file7 (DOCX 16 KB)

Supplementary file8 (DOCX 14 KB)

## Data Availability

10 × scRNA-seq data of PDAC samples were downloaded from the China National Center for Bioinformation (https://ngdc.cncb.ac.cn/). All data and R script in this study are available from the corresponding author upon reasonable request. All authors read and approved the final manuscript. Publicly available datasets were analyzed in this study, these can be found in The Cancer Genome Atlas (https://portal.gdc.cancer.gov/), International Cancer Genome Consortium (https://dcc.icgc.org), and Gene Expression Omnibus (GSE62165, GSE71989, GSE16515, GSE91035, GSE62452, GSE57495).
