# Supplementary material for: The crucial prognostic signaling pathways of pancreatic ductal adenocarcinoma were identified by single-cell and bulk RNA sequencing data
Source: Hum Genet. 2024 Mar 25;143(9-10):1109–29. doi: 10.1007/s00439-024-02663-4 (PMC11485037; doi:10.1007/s00439-024-02663-4)
Supplement: Supplementary file 1 — Supplementary file1 (DOCX 1173 KB) [file 439_2024_2663_MOESM1_ESM.docx]

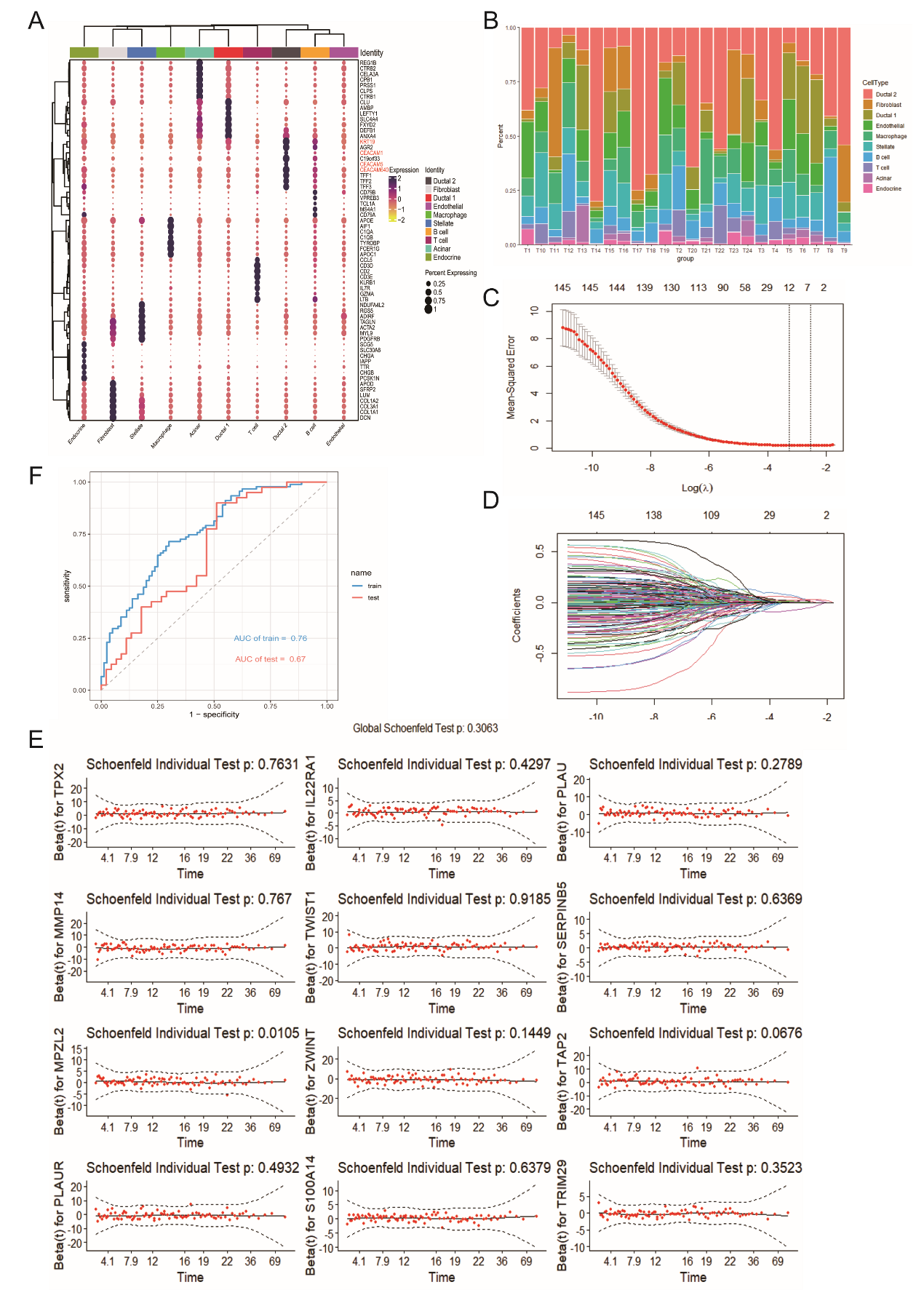


**Supplementary Figure 1.** Identification of DEGs based on scRNA-seq and GEO datasets, and establishing prognostic model. **(A)** Bubble plot showing the marker genes across all clusters. Size of dots represents the proportion of cells expressing a particular marker, and intensity of color indicates the average expression level. **(B)** Proportions of different cell types among different patients. **(C-D)** Variable selection using LASSO regression, the first dashed line showing the cutoff value we selected, indicating minimal deviance (C), and the correlation between coefficients and the number of variable (D). **(E)** The 12-gene proportional hazards assumption was tested using Schoenfeld residuals. **(F)** Receiver operating characteristic (ROC) curves of the multivariate Cox regression model in the training set and the internal validation set.
