# Supplementary material for: The crucial prognostic signaling pathways of pancreatic ductal adenocarcinoma were identified by single-cell and bulk RNA sequencing data
Source: Hum Genet. 2024 Mar 25;143(9-10):1109–29. doi: 10.1007/s00439-024-02663-4 (PMC11485037; doi:10.1007/s00439-024-02663-4)
Supplement: Supplementary file 2 — Supplementary file2 (DOCX 1060 KB) [file 439_2024_2663_MOESM2_ESM.docx]

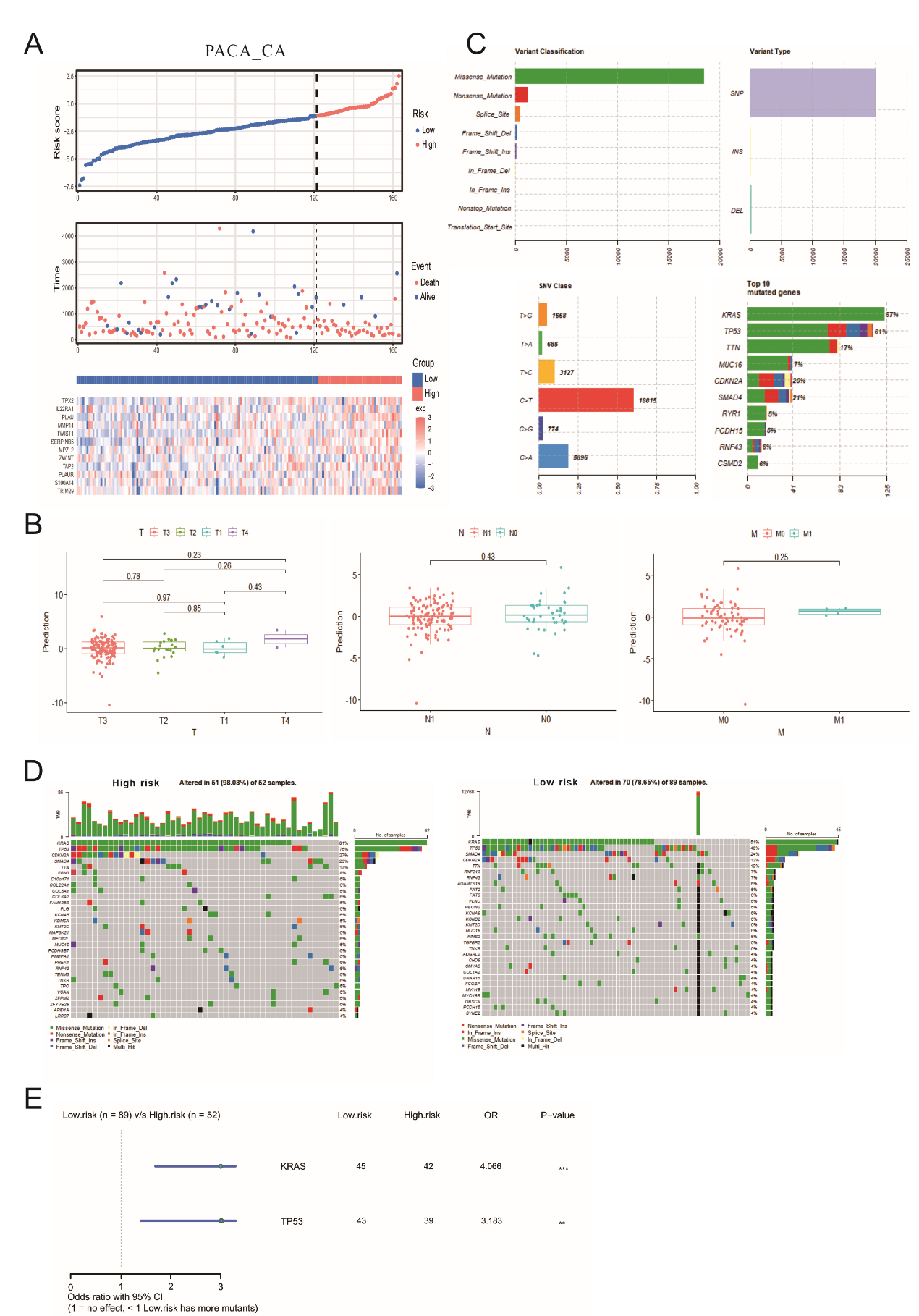


**Supplementary Figure 2.** Clinical relevance, nomogram and mutation landscape between high- and low-risk groups. **(A)** Risk plots to illustrate the survival status of each sample and signature genes expression heatmaps in the PACA_CA cohort. **(B)** Boxplot of the correlation between tumor TNM and model prognostic prediction score. **(C-D)** PDAC of TCGA cohort tumor genes mutation landscape and the waterfall plots summarize the gene mutation landscape in high- and low-risk groups. **(E)** Multivariate logistic regression analyses were used to select risk factors of the mutation genes of PDAC.
