# Supplementary material for: The crucial prognostic signaling pathways of pancreatic ductal adenocarcinoma were identified by single-cell and bulk RNA sequencing data
Source: Hum Genet. 2024 Mar 25;143(9-10):1109–29. doi: 10.1007/s00439-024-02663-4 (PMC11485037; doi:10.1007/s00439-024-02663-4)
Supplement: Supplementary file 3 — Supplementary file3 (DOCX 393 KB) [file 439_2024_2663_MOESM3_ESM.docx]

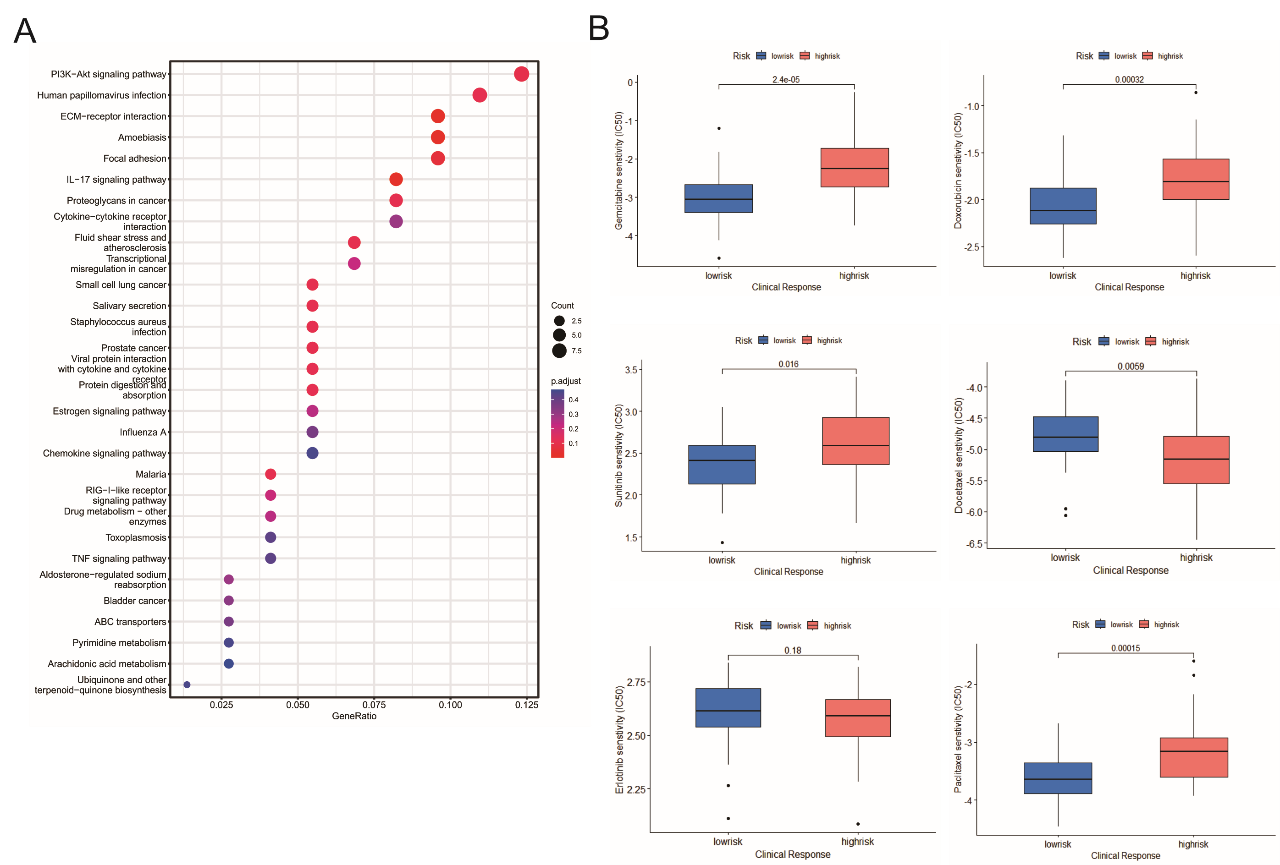


**Supplementary Figure 3.** Differential gene analysis and chemotherapy drug sensitivity between high-risk group and low-risk group. **(A)** KEGG analysis of differentially expressed genes between high- and low-risk groups. **(B)** Drug sensitivity analysis of GSE62452 cohort between high and low risk groups. Estimated IC50 for Docetaxel, Gemcitabine, Paclitaxel, Doxorubicin, Sunitinib, and Erlotinib.
