# Supplementary material for: The crucial prognostic signaling pathways of pancreatic ductal adenocarcinoma were identified by single-cell and bulk RNA sequencing data
Source: Hum Genet. 2024 Mar 25;143(9-10):1109–29. doi: 10.1007/s00439-024-02663-4 (PMC11485037; doi:10.1007/s00439-024-02663-4)
Supplement: Supplementary file 4 — Supplementary file4 (DOCX 2721 KB) [file 439_2024_2663_MOESM4_ESM.docx]

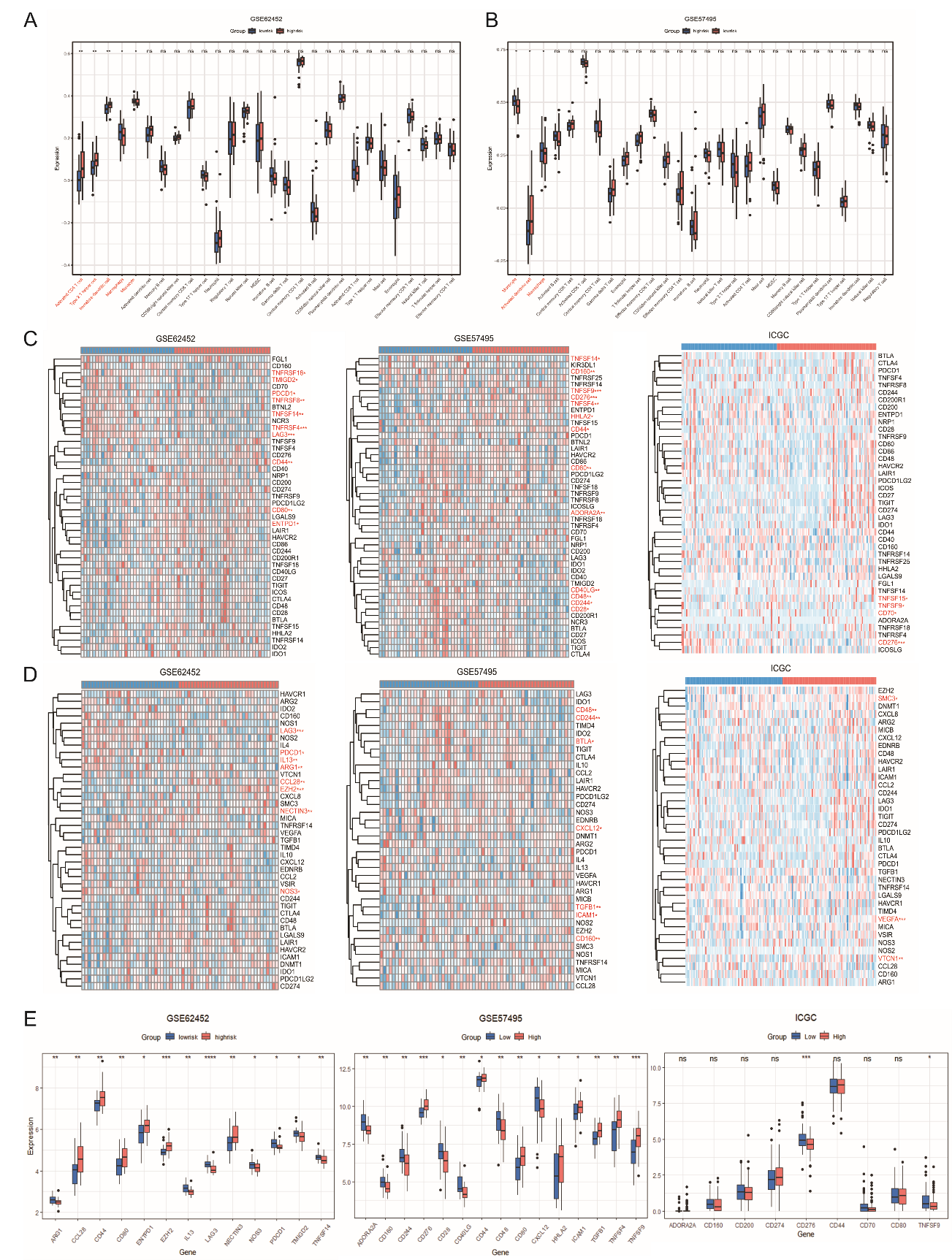


**Supplementary Figure 4.** Immune landscape analysis of the GEO cohort. **(A-B)** Immune cells infiltration levels in the low- and high-risk groups estimated by ssGSEA in GSE62452 and GSE57495 cohorts. **(C)** Heatmap of Cancer-Immunity Cycle-related genes expression patterns between the high- and low-risk groups of GSE62452, GSE57495, and ICGC cohorts. **(D)** Heatmap of immune checkpoint genes expression patterns in the high- and low-risk groups in the GSE62452, GSE57495, and ICGC cohorts. **(E)** Boxplot of representative immune-related genes expression between high- and low-risk groups in the GSE62452, GSE57495, and ICGC cohorts.
