# Supplementary material for: The crucial prognostic signaling pathways of pancreatic ductal adenocarcinoma were identified by single-cell and bulk RNA sequencing data
Source: Hum Genet. 2024 Mar 25;143(9-10):1109–29. doi: 10.1007/s00439-024-02663-4 (PMC11485037; doi:10.1007/s00439-024-02663-4)
Supplement: Supplementary file 5 — Supplementary file5 (DOCX 2186 KB) [file 439_2024_2663_MOESM5_ESM.docx]

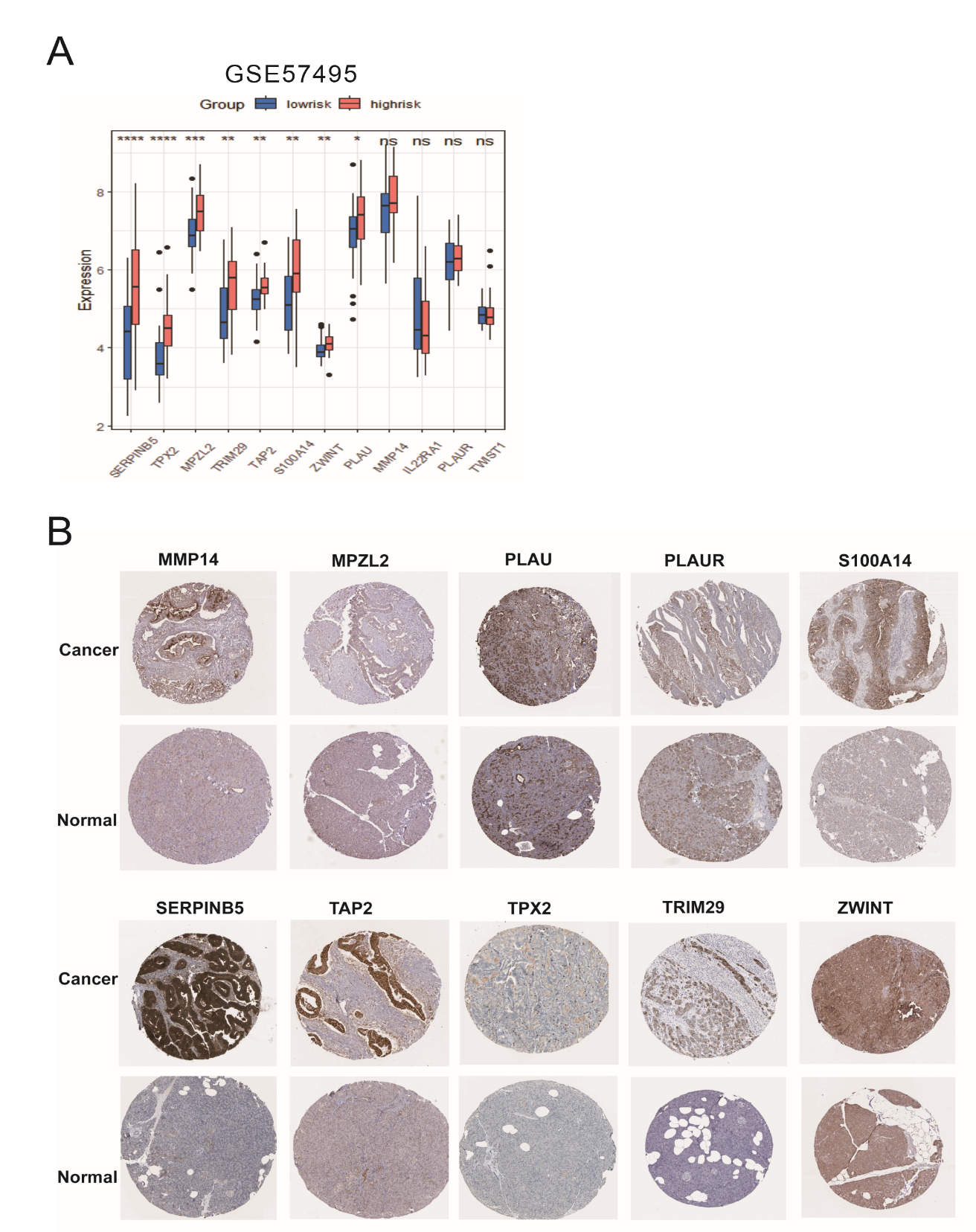


**Supplementary Figure 5.** Expression analysis, PPI network identification and cytological experiment verification of prognostic related molecules. **(A)** The boxplot shows the expression levels of signature genes in GSE57495 cohorts. **(B)** Immunohistochemistry staining of twelve signature genes in the pancreas normal tissue and pancreas cancer tissue.
