# Supplementary material for: The crucial prognostic signaling pathways of pancreatic ductal adenocarcinoma were identified by single-cell and bulk RNA sequencing data
Source: Hum Genet. 2024 Mar 25;143(9-10):1109–29. doi: 10.1007/s00439-024-02663-4 (PMC11485037; doi:10.1007/s00439-024-02663-4)
Supplement: Supplementary file 6 — Supplementary file6 (DOCX 560 KB) [file 439_2024_2663_MOESM6_ESM.docx]

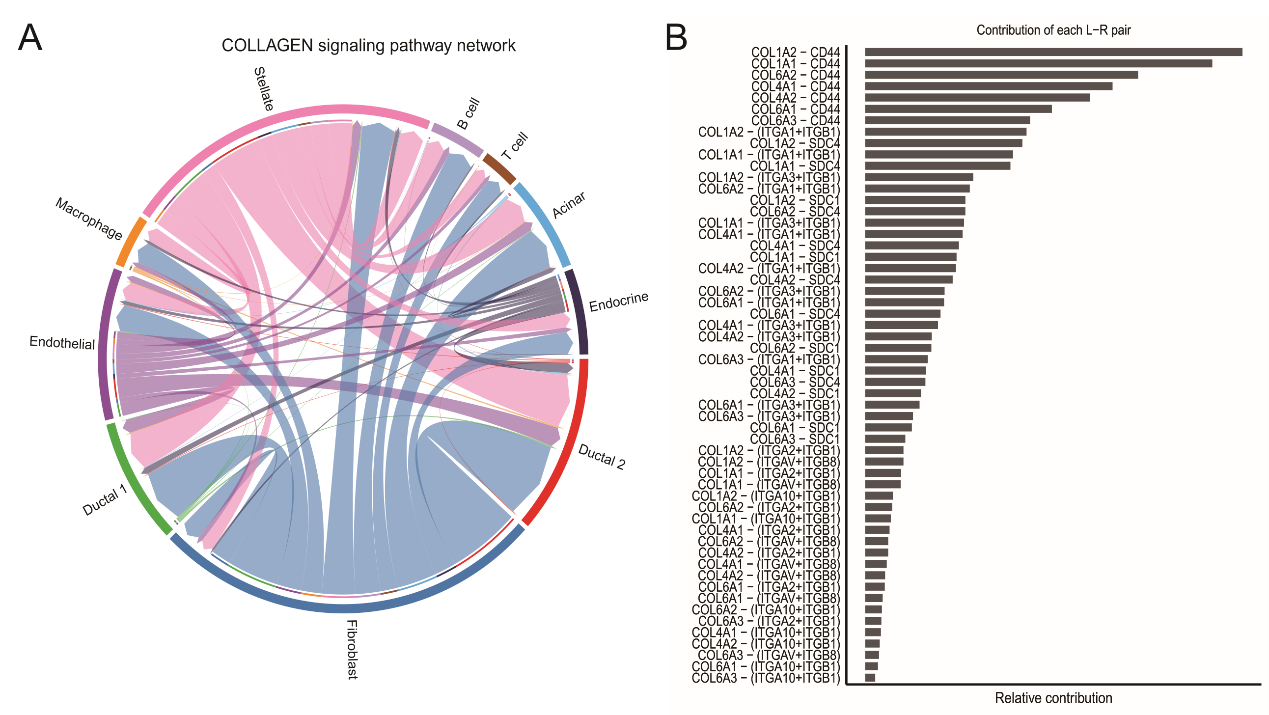


**Supplementary Figure 6.** Communication analysis of cell subsets. **(A)** Cell-cell communication network identified of collagen signaling pathway network. **(B)** Contribution values of paired receptors and ligands between cells in the collagen pathway in the pathway.
