# Supplementary material for: The crucial prognostic signaling pathways of pancreatic ductal adenocarcinoma were identified by single-cell and bulk RNA sequencing data
Source: Hum Genet. 2024 Mar 25;143(9-10):1109–29. doi: 10.1007/s00439-024-02663-4 (PMC11485037; doi:10.1007/s00439-024-02663-4)
Supplement: Supplementary file 7 — Supplementary file7 (DOCX 16 KB) [file 439_2024_2663_MOESM7_ESM.docx]

Suppletory Table 1. Table of data information were used for the article

|  | **TCGA_PAAD**  **(n = 177)** | **PACA_CA**  **(n = 167)** | **PRJCA001063**  **(n = 45)** | **GSE62165**  **(n = 131)** | **GSE71989**  **(n = 22)** | **GSE16515**  **(n = 52)** | **GSE91035**  **(n = 50)** | **GSE62452**  **(n = 130)** | **GSE57495**  **(n = 63)** |
| --- | --- | --- | --- | --- | --- | --- | --- | --- | --- |
| **Platform** | HiSeq  (RNA-seq) | HiSeq  (RNA-seq) | 10X  (scRNA-seq) | GPL13667  (Gene chip) | GPL570  (Gene chip) | GPL570  (Gene chip) | GPL22763  (Gene chip) | GPL6244  (Gene chip) | GPL15048  (Gene chip) |
| **Gender** | | | | | | | | | |
| Male | 97 | 90 | 18 | NA | NA | 34 | NA | NA | NA |
| Female | 80 | 76 | 23 | NA | NA | 18 | NA | NA | NA |
| Unknown | 0 | 0 | 3 | NA | NA | 0 | NA | NA | NA |
| **Age (years)** | | | | | | | | | |
| Median (range) | 65 (36-89) | 67 (35-90) | 63.5 (59-71) | NA | NA | 68.5 (49-84) | NA | NA | NA |
| **Stage** | | | | | | | | | |
| I | 21 | 51 | 10 | 8 | NA | NA | NA | 7 | 13 |
| IIA | 28 | 27 | 5 | 30 | NA | NA | NA | 18 | 17 |
| IIB | 118 | 56 | 10 | 62 | NA | NA | NA | 66 | 33 |
| III | 3 | 7 | 3 | 5 | NA | NA | NA | 26 | 0 |
| IV | 4 | 3 | 0 | 13 | NA | NA | NA | 13 | 0 |
| Others | 3 | 23 | 13 | 13 | NA | NA | NA | 0 | 0 |
| **Tumor** | 173 | 167 | 24 | 118 | 13 | 36 | 25 | 69 | 63 |
| **Decreased** | 130 | 133 | NA | 13 | NA | NA | NA | 49 | 42 |
| **Location** | | | | | | | | | |
| Head | 129 | NA | 11 | 93 | NA | NA | NA | NA | NA |
| Body | 15 | NA | 9 | 15 | NA | NA | NA | NA | NA |
| Tail | 14 | NA | 4 | 10 | NA | NA | NA | NA | NA |
| Others | 19 | NA | 17 | 13 | NA | NA | NA | NA | NA |
