# Supplementary material for: The crucial prognostic signaling pathways of pancreatic ductal adenocarcinoma were identified by single-cell and bulk RNA sequencing data
Source: Hum Genet. 2024 Mar 25;143(9-10):1109–29. doi: 10.1007/s00439-024-02663-4 (PMC11485037; doi:10.1007/s00439-024-02663-4)
Supplement: Supplementary file 8 — Supplementary file8 (DOCX 14 KB) [file 439_2024_2663_MOESM8_ESM.docx]

Suppletory Table 2. List of primer sequence information for real-time PCR

| **Primer names** | **Primer sequences (5' to 3')** |
| --- | --- |
| TPX2 (human)-F | AGGGCCTTTCTGGTTCTCTAGT |
| TPX2 (human)-R | TGCCTTATGCACCAGTTCTC |
| IL22RA1 (human)-F | CTCCCAACTCCCTGAACGTC |
| IL22RA1 (human)-R | AGGTGATCTCGGACAGGCTA |
| PLAU (human)-F | CGAGCCGCCGTCTAGC |
| PLAU (human)-R | GTTCATTGCTGCCTTTGGAGT |
| MMP14 (human)-F | TCCAGCAACTTTATGGGGGT |
| MMP14 (human)-R | TTCCCGTCACAGATGTTGGG |
| TWIST1 (human)-F | TACGCCTTCTCGGTCTGGA |
| TWIST1 (human)-R | TCTCTGGAAACAATGACATCTAGG |
| SERPINB5 (human)-F | GGCTTTTGCCGTTGATCTGT |
| SERPINB5 (human)-R | TTTCAAAATGAAGAACCTGTCCAA |
| MPZL2 (human)-F | GGGGACCTGAGCAGTTTGTAT |
| MPZL2 (human)-R | TCCAGAGAAGGATGGAGGCA |
| ZWINT (human)-F | AGGACACTGCTAAGGGTCTCG |
| ZWINT (human)-R | GCCTCTACGTGCTCCCTGTA |
| TAP2 (human)-F | TGGACGCGGCTTTACTGTG |
| TAP2 (human)-R | GCAGCCCTCTTAGCTTTAGCA |
| PLAUR (human)-F | GAGAGAAGACGTGCAGGGAC |
| PLAUR (human)-R | GCCCCAAGAGGCTGGGA |
| S100A14 (human)-F | CCATCTCATGCCGAGCAACT |
| S100A14 (human)-R | CCACAGTCTCTCCCCAACAC |
| TRIM29 (human)-F | CCCATCCGGGACTTTGAGG |
| TRIM29 (human)-R | TGACAGCTCCGTCTCCTTCT |
| beta actin (human)-F | CATGTACGTTGCTATCCAGGC |
| beta actin (human)-R | CTCCTTAATGTCACGCACGAT |
| COL1A1 (human)-F | GAGGGCCAAGACGAAGACATC |
| COL1A1 (human)-R | CAGATCACGTCATCGCACAAC |
| COL1A2 (human)-F | GTTGCTGCTTGCAGTAACCTT |
| COL1A2 (human)-R | AGGGCCAAGTCCAACTCCTT |
| SDC1 (human)-F | CTGCCGCAAATTGTGGCTAC |
| SDC1 (human)-R | TGAGCCGGAGAAGTTGTCAGA |
| SDC4 (human)-F | GGACCTCCTAGAAGGCCGATA |
| SDC4 (human)-R | AGGGCCGATCATGGAGTCTT |
| ITGA1 (human)-F | GCTCCTCACTGTTGTTCTACG |
| ITGA1 (human)-R | CGGGCCGCTGAAAGTCATT |
| ITGA10 (human)-F | AACATCACCCACGCCTATTCC |
| ITGA10 (human)-R | GTTGGTAGTCACCTAAGTGGC |
| ITGB1 (human)-F | CCTACTTCTGCACGATGTGATG |
| ITGB1 (human)-R | CCTTTGCTACGGTTGGTTACATT |
| ITGB8 (human)-F | ACCAGGAGAAGTGTCTATCCAG |
| ITGB8 (human)-R | CCAAGACGAAAGTCACGGGA |
